# Initiating Sensory Science Research in Space Conditions—Current Practices and Future Perspectives

**DOI:** 10.1111/1541-4337.70241

**Published:** 2025-08-03

**Authors:** Thejani Prabodha, Lukas Danner, Gail N. Iles, Martina Heer, Isabelle Mack, Charles Brennan, Julia Low

**Affiliations:** ^1^ Sensory and Consumer Science Research Group, School of Science RMIT University, Bundoora West Campus Bundoora Victoria Australia; ^2^ Agriculture and Food Commonwealth Scientific and Industrial Research Organisation (CSIRO) Werribee Victoria Australia; ^3^ Space Physics Group, School of Science RMIT University Melbourne Victoria Australia; ^4^ IU International University of Applied Sciences Erfurt Germany; ^5^ Department of Internal Medicine VI University Hospital Tübingen Tübingen Germany; ^6^ STEM College RMIT University Melbourne Victoria Australia

**Keywords:** astronaut diet, confined environments, extraterrestrial habitats, isolation and confinement, lunar gravity, Mars missions, microgravity, sensory adaptation, sensory perception, space analogs, space flavor, space food, space nutrition, spaceflight

## Abstract

Space exploration continues to drive technological and scientific advancements, benefiting both space and Earth. This expanding sector, including commercial space stations, deep‐space missions, and space tourism, presents unique challenges and opportunities for food researchers. A critical issue is the limited understanding of how space conditions such as microgravity, isolation, and confinement affect sensory perception, impacting astronauts’ nutritional intake and psychological well‐being. This review based on a structured literature search strategy integrates existing data on flavor perception during spaceflight missions (*n* = 16), identifying sensory changes, altered flavor preferences, and cravings linked to space‐related stressors. It also evaluates space analogs and simulations related to sensory research (*n* = 29), assessing their applicability for studying food perception in space conditions. It identified consistent sensory changes, including diminished taste sensitivity, increased preference for spicy or umami‑rich foods, and intense flavors. However, research in this area remains limited, with small sample sizes, inconsistent methodologies, and lack of comprehensive guidelines on applying analogs and simulations in sensory studies. These gaps raise challenges for researchers, particularly those without access to space analogs or simulations, in designing space‐related studies. Critically analyzing the findings, the review identifies gaps in understanding the complex interactions between environmental, physiological, and sensory factors. It also outlines potential methodological pathways to strengthen future evidence generation, such as implementing ecologically valid research approaches and validated analog designs, including combined use of multiple analog environments to better replicate the multidimensional sensory context. Addressing these challenges will support astronaut well‐being and foster innovations that benefit both space and Earth‐based applications.

## 1 Introduction

### 1.1 The Multifaceted Role of Food and Flavor Perception in Space Missions

Nutritional requirements and food systems for long‐duration space missions are crucial to crew safety and mission success, just as the spacecraft's mechanical systems are (Douglas et al. 2020). Despite advancements since the launch of the International Space Station (ISS) in 1998, it remains unclear why space travelers consistently consume only about 80% of their daily energy needs (Smith et al. 2005). In the second edition of *Human Adaptation in Spaceflight: The Role of Food and Nutrition* (Smith et al. 2020) by the National Aeronautics and Space Administration (NASA), microgravity, radiation, isolation and confinement, environmental, and time factors are identified as key stressors impacting crew nutritional requirements. Balanced nutrition and healthy eating behavior are essential for counteracting these stressors and maintaining optimal human functioning during space missions (Bergouignan et al. 2016; Cena et al. 2003; Heer et al. 2000; Pandith et al. 2023; Pittia et al. 2023; Smith et al. 2021). Despite significant improvements in the space food system over the past 50 years, which have enhanced meal palatability for Low Earth Orbit (LEO) crews, these advancements fall short for long‐duration missions, such as the proposed 3‐year journey to Mars (Vodovotz et al. 2000). The challenges of preventing weight loss and ensuring adequate nutrition on these extended missions present serious challenges to both physical and psychological well‐being.

The importance of food in space extends far beyond meeting basic energy requirements (Cooper et al. 2011); it becomes a crucial source of psychological comfort and gratification, especially on prolonged missions where other forms of personal satisfaction are limited. As reported in Stuster's (2016) analysis of the *Journals* experiment, which documented astronauts’ daily experiences aboard the ISS, food consistently emerged as a primary subject of interest and discussion. Stuster (2016) noted that “food assumes added importance when access to friends, family, leisure pursuits and other normal sources of gratification are denied,” emphasizing that food's role in maintaining morale is similarly observed in other isolated and confined environments such as oil rigs, Antarctic research stations, and submarines. Thus, understanding eating behavior in such settings requires more than addressing physiological needs—it demands an exploration of the broader social and psychological roles food plays.

The space environment may significantly alter flavor perception and food palatability, which can, in turn, impact nutritional well‐being (Taylor et al. 2021; Taylor et al. 2020). Beyond the obvious reduction in gravity, the spacecraft environment is highly confined and cluttered, presenting unique eating challenges. Taylor and colleagues (2020) highlighted numerous internal and external factors within the spacecraft that could influence flavor perception (e.g., water quality, eating environment, stress). Flavor perception itself is inherently multisensory, involving the integration of taste, smell, texture, and trigeminal sensations. These sensory inputs integrate within the individual and are further influenced by the external environment (Holthuysen et al. 2017; Low et al. 2024; Schouteten et al. 2024; Spence et al. 2019; Wang et al. 2019), creating a unique perceptual experience for each person and every eating occasion. The five basic senses—touch, taste, smell, sight, and hearing—work together to form a multisensory perception of food, contributing significantly to eating enjoyment (Newman et al. 2023; Zampini and Spence 2010). Consequently, any disruption to one of these senses, such as reduced smell sensitivity in microgravity, as well as food presentation and consumption methods, may dramatically alter the perception and acceptance of food, highlighting the need for sensory adaptive food systems that can accommodate such environmental effects.

Although the atmospheric pressure aboard the ISS is maintained at a level comparable to sea level on Earth (OCHMO 2023), astronauts’ sensory experiences reveal a more complex interaction. For instance, astronaut Scott Parazynski likened the experience of having a stuffy nose in LEO to having a cold or allergies, which dulls the senses of smell and taste (Romanoff 2009). This highlights the correlation between nasal congestion and diminished flavor perception (Akerlund et al. 1995; Eccles et al. 1989; Pellegrino et al. 2017). Additionally, space travelers have reported changes in food preferences during missions, such as developing a liking for foods like shrimp cocktails with freeze‐dried horseradish, which they might not have enjoyed on Earth, while finding other items, such as coffee, less appealing compared to their usual experiences (Bourland and Vogt 2010; Kerwin and Seddon 2002). As discussed by Olabi and colleagues (2002), coffee's complex aroma, which relies on over 500 chemical odorants (Angeloni et al. 2021; Czerny and Grosch 2000; Spiller 2019), is likely muted in space due to nasal congestion and the absence of certain odorants, resulting in a more bitter taste. Despite these reported changes, quantitative data on the extent of flavor perception are scarce due to the limited sensory evaluations conducted during missions, with a typical crew size of around six members (Salotti et al. 2014). While previous reviews have addressed physiological and psychological responses to space environments more broadly, a detailed examination of how flavor perception is affected across real spaceflights, analogs, and digital simulations remains lacking. This review bridges this gap by examining flavor‐related responses across diverse methods and platforms to identify both patterns and methodological limitations. This review not only consolidates current findings but also critically evaluates methodologies used across diverse environments, offering practical guidance for designing future sensory studies in space‐relevant contexts. Understanding flavor perception under space conditions has direct implications for maintaining astronaut health, developing sensory countermeasures, and translating findings to address sensory challenges in extreme or isolated environments on Earth.

Given these sensory challenges, developing realistic Earth‐based simulations of space conditions is essential for designing nutritious and palatable foods for long space missions. Key reviews in the field (Olabi et al. 2002; Taylor et al. 2020) have highlighted numerous factors that affect dietary intake in space, underscoring the need to study these elements individually or in combination to better understand their impact on flavor perception. By simulating specific space‐related conditions in laboratory settings, researchers can gain insights into how environmental and psychological factors alter food preferences and sensory experiences. While challenging, designing setups that mimic space's unique conditions represents a crucial step toward enhancing dietary intake and satisfaction for astronauts during spaceflight.

### 1.2 Structure, Aims, and Objectives

This narrative review based on a structured literature search strategy examines flavor perception research within spaceflight missions and Earth‐based analogs, aiming to explore how these platforms can contribute to understanding food sensory experiences in space. Throughout this review, we use the currently accepted definitions of *simulated microgravity* to refer to tools such as clinostats and random positioning machines, and *microgravity analogs* to refer to human‐rated methods such as head‐down bed rest and dry immersion (Ferranti et al. 2020; Oluwafemi and Neduncheran 2022). We also include *microgravity platforms* like parabolic flights and orbital missions, which provide actual microgravity exposure. Table 1 summarizes the differences utilizing definitions provided by Ferranti et al. (2020) and Oluwafemi and Neduncheran (2022). In addition, this review considers *isolation and confinement analogs* that simulate the psychological and operational stressors of spaceflight, and *digital simulations* which replicate the environmental context of space without mimicking gravitational effects.

**TABLE 1:** Categorization of microgravity‐related facilities and equipment listed as simulations, analogs, or platforms.

Rating	Simulated microgravity^a^	Microgravity analog^a^	Microgravity platform^a^
Human rated	—	Bed rest, dry immersion, wet immersion, neutral buoyancy, unilateral lower limb suspension (ULLS)	Parabolic flight, sub‐orbital rocket flight, orbital flight
Nonhuman rated	Clinostat, random positioning machine (RPM), rotating wall vessel (RWV), diamagnetic levitation	—	Sounding rocket flight

^a^ Definitions adapted from Oluwafemi and Neduncheran (2022) and Ferranti et al. (2020).

**FIGURE 1:**
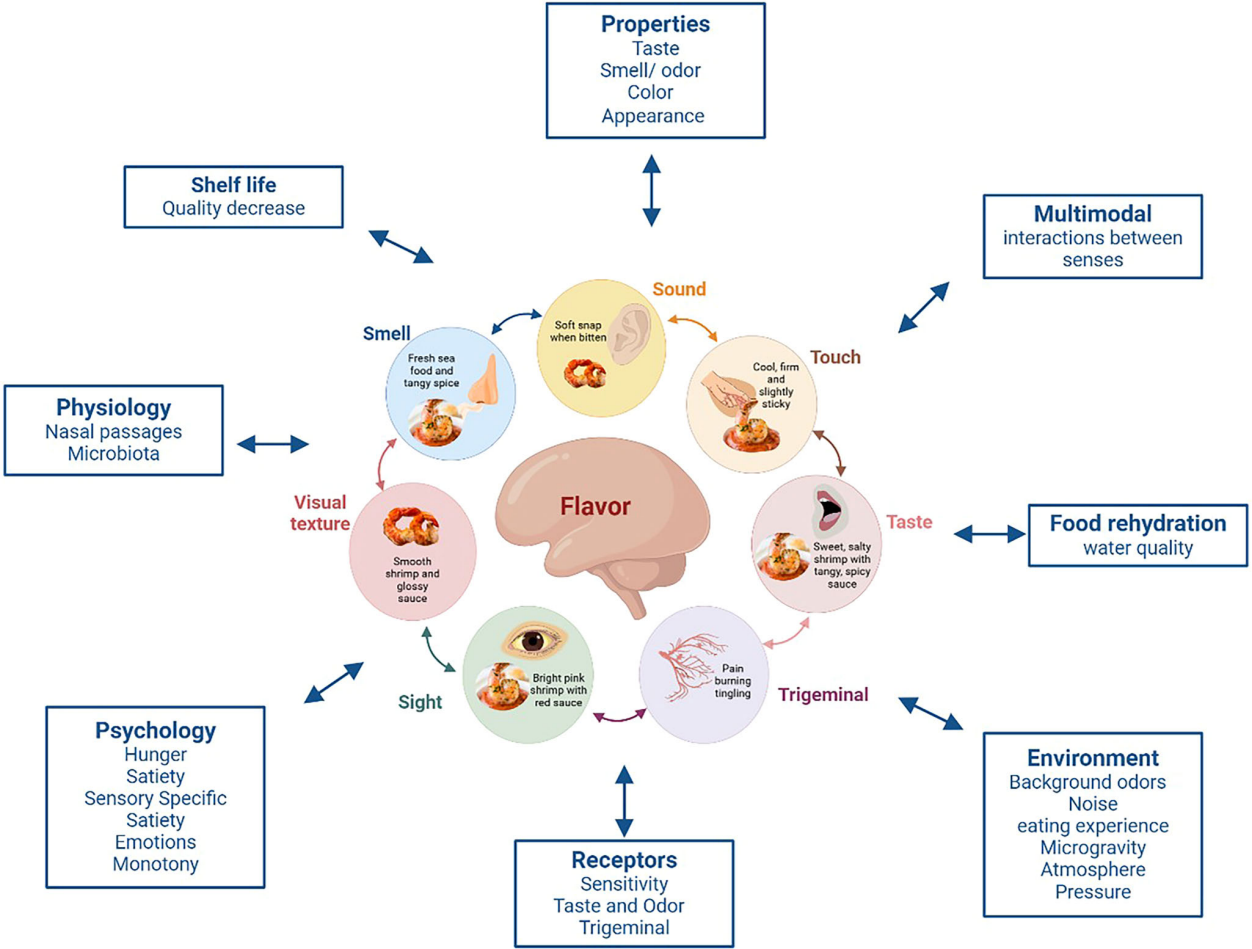
Adapted schematic representation illustrating how the five core human senses—(1) sight (including visual texture); (2) smell; (3) sound; (4) touch; (5) taste along with the trigeminal senses—interact to shape overall flavor perception of a shrimp (prawn) cocktail in space, which is known popular food choice among space travelers. Adapted from Newman et al. (2023) and Taylor et al. (2020).

While recent reviews have addressed space analogs in life sciences broadly (Oluwafemi and Neduncheran 2022), their specific application to flavor perception remains underexplored. Therefore, this review aims to comprehensively examine methods and tools used to assess flavor perception specifically in space missions (Section 3). By exploring this information, we aim to identify any consistent changes in flavor perception during space missions, which could inform future research in prolonged, confined environments. In this review, flavor perception is defined as the interaction between the five core human senses—taste, smell (including orthonasal and retronasal olfaction), sight (color, visual texture), sound, and touch—and trigeminal senses (Zampini and Spence 2010; Figure 1 adapted from Newman et al. [2023]). The second part of the review focuses on identifying the available space analogs and simulation methodologies used in space life science studies, with a particular emphasis on their applicability for studying flavor perception and eating experience (Section 4). This includes a discussion of the relative merits (limitations and benefits) of key methodologies and research gaps. Recommendations and conclusive remarks were summarized in Section 5. The background context of current space travel conditions, including the challenges associated with physiological changes during space travel, is provided in Supporting Information A. A narrative review approach was selected, supported by a structured literature search strategy, to map the current range of space analogs and simulations applicable to flavor perception research. This methodology allows for a broader examination of existing methods, helping to highlight gaps in knowledge and identify tools that could be adapted for studying sensory experiences in space.

## 2 Methods

Peer‐reviewed studies published from the first identified relevant paper in January 1974 to November 2024 (including in‐press articles at the time of analysis) were eligible for inclusion in the review, sourced from PubMed, Google Scholar, Scopus, and Web of Science databases. The following inclusion criteria were applied:

1. Peer‐reviewed journals in English (translated articles were considered if relevant);
2. Studies investigating flavor perception (taste, smell, visual, sound, touch, trigeminal) that can be directly simulated through stimuli impacting food perception in spaceflight missions, analogs, and simulations. “Direct experience” included tasting foods or prototype taste stimuli, sniffing food‐related odorants, and viewing food‐related media (videos, images). Studies that measured sensory characteristics (e.g., intensity, functionality) and affective responses were also included;
3. Studies involving the indirect sensory evaluation of food such as eating behavior in space analogs and simulations (e.g., human cognitive performance related to food, ingestive behavior such as food cravings). These studies provide insight into the applicability of analog methods to flavor perception.

**FIGURE 2:**
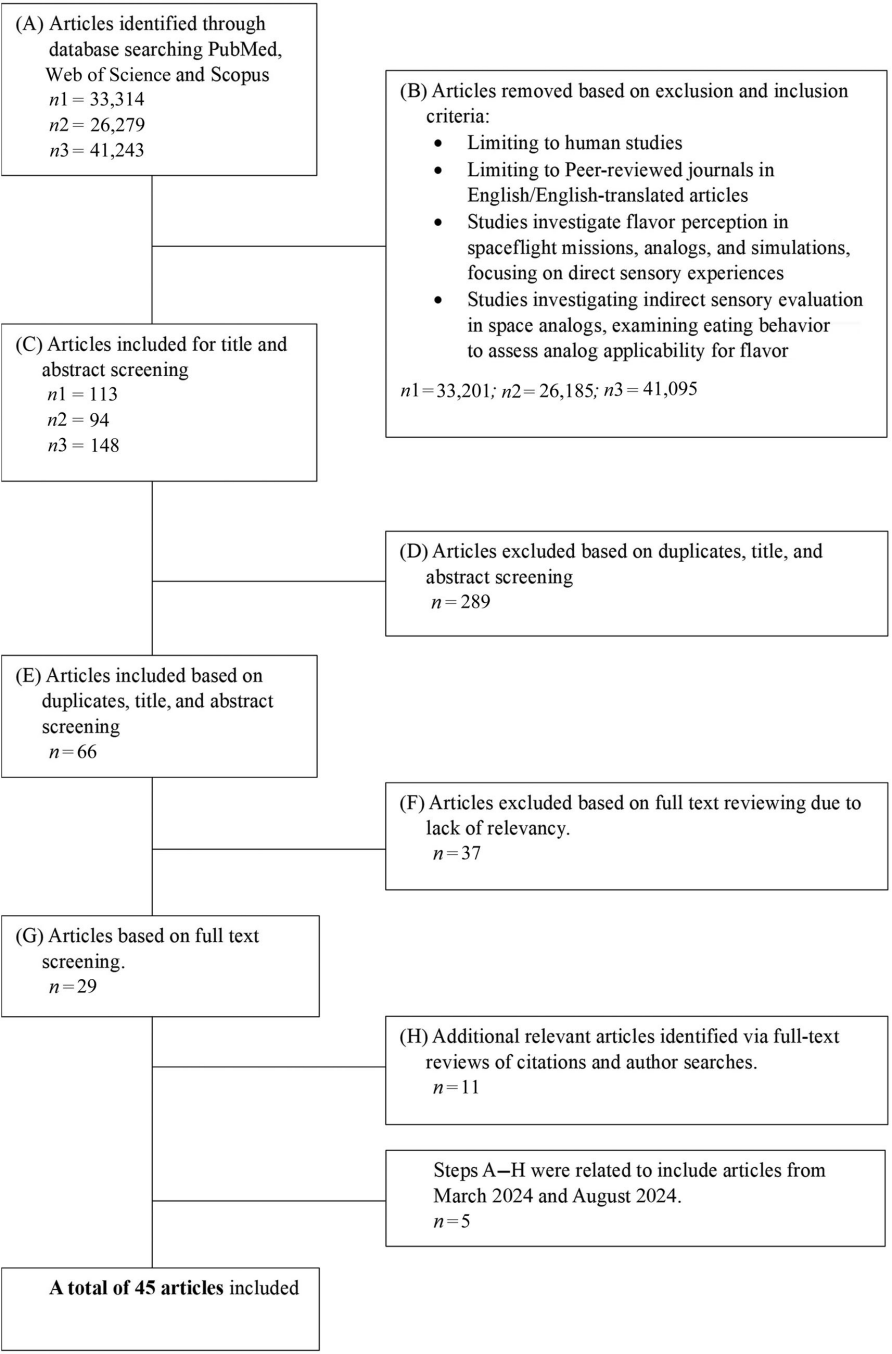
Schematic representation of literature search and selection procedure. Specific keyword combinations included “space,” “food*,” “sensory,” “microgravity,” “spaceflight OR space flight,” “taste,” “smell,” “flavour OR flavor,” “chemosensory,” “perception,” “analog*,” “simulation,” “isolat*,” and “confine*.” Specific keyword combinations included (Space OR spaceflight OR space flight OR microgravity) AND food*; (Space OR spaceflight OR space flight OR microgravity) AND (taste OR smell OR flavour OR flavor); (Space OR spaceflight OR space flight OR microgravity) AND (chemosensory OR perception); Space AND (analog* OR simulation) AND food*; Space AND (analog* OR simulation) AND (taste OR smell OR flavour OR flavor); Space AND (analog* OR simulation) AND (chemosensory OR perception); and Space AND (analog* OR simulation) AND (isolat* OR confine*).

Two independent literature searches were conducted in June 2023, with slight modifications to keywords to ensure the inclusion of relevant studies. Additional screenings were performed in March 2024 and August 2024 to include any newly published studies. The search used the following keywords in various combinations: “space,” “food*,” “sensory,” “microgravity,” “spaceflight OR space flight,” “taste,” “smell,” “flavour OR flavor,” “chemosensory,” “perception,” “analog*,” “simulation,” “isolat*,” and “confine*.” Additional articles were identified through full‐text reviews of citations and searches based on first author names. A schematic representation of the search strategy can be found in Figure 2. A total of 45 articles were identified.

## 3 Results and Discussions: Understanding Flavor Perception in Spaceflight Conditions

A total of 16 articles were identified that explored how spaceflight conditions affect flavor perception. These included four quantitative studies and 12 qualitative observations captured during space missions. While quantitative data collection on sensory perception related to space conditions was primarily conducted in the 1970s and 1980s (e.g., Soyuz 30–31 missions, Skylab 4, and STS‐41‐G), no such studies have been conducted aboard the ISS, with available data limited to qualitative reports. This highlights the challenge in evaluating the long‐term impact of space conditions on flavor perception. This persistent absence of in‐flight quantitative research not only limits understanding of how flavor perception evolves under space conditions but also hinders the development of evidence‐based countermeasures for future long‐duration missions. Given the central role of food in sustaining both nutrition and psychological well‐being, the lack of robust sensory data represents a critical gap that future space programs must prioritize through controlled, space‐compatible sensory evaluations. While this section focuses on summarizing current findings, the authors recommend that future studies employ standardized, space‐compatible psychophysical methods and repeated‐measure designs to systematically track flavor perception changes over time. Broader recommendations and methodological considerations are further discussed in Section 4.

This section presents results and discussion in two parts: (1) subjective experiences of flavor perception during spaceflight (Section 3.1) and (2) quantitative data on flavor perception in spaceflight studies (Section 3.2). Collectively, the discussion below offers insights into sensory adjustments to space environments and reveals behavioral adaptation among astronauts to maintain food pleasure.

### 3.1 Flavor Perception During Spaceflights—Subjective Experiences

The effects of space conditions on flavor perception experienced by crew members are summarized in Table 2 and divided into three key themes: changes in sensory perception; behavioral strategies for enhancing food enjoyment; and appetite and cravings. Each theme highlights potential sensory changes and adaptive eating behaviors, suggesting avenues for improving food experiences in space. The studies published between 1963 and 2020 primarily relied on astronaut debriefs, mission logs, and informal interviews.

**TABLE 2:** Subjective experiences related to flavor perception during spaceflights categorized by themes.

Author (year)	Space mission	Theme(s)	Subjective experiences
Rambaut et al. 1977; Baranski et al. 1983	Subjective reports of Soviet and American crew members	Sensory Perception (Taste−)/Others	Perceived overall blandness and attenuation in taste sensitivity, with some crew members reporting an unpleasant taste in the oral cavity.
Oberg 1981	Vostok 6 (1963)	Appetite/Strategies for Maintaining Food Pleasure (trigeminal)	Decreased appetite for sweets, with a strong desire for spicy and pungent flavors.
Campton and Benson 1983	Apollo mission (1968–1969)	Sensory Perception (Taste−)/Appetite	Altered taste, texture, and appeal. Sausage patties found unappetizing when compared to “coarse granulated rubber.”
Rambaut et al. 1977; Kerwin and Seddon 2002	Skylab 2 mission (1973)	Strategies for Maintaining Food Pleasure (trigeminal)	Crewmembers added spices to their food supply to enhance flavor. Reduced taste perception, potentially due to monotony, and tested condiments to improve palatability.
Kerwin and Seddon 2002; Da Silva et al. 2002; Johnston and Dietlein 1977; Turner and Sanford 1974	Skylab 3 mission (1973)	Food Preferences/Sensory Perception (Taste−)/Strategies for Maintaining Food Pleasure (trigeminal)/Others	Perceived less taste in foods while in orbit than on the ground. This was thought to be due to the monotony of the diet rather than a physiological change. Dietary preference for higher carbohydrates in relation to fat. Use of spices and condiments increases the palatability.
Nicogossian 1977; Da Silva et al. 2002	Apollo‐Soyuz flight (1975)	Food Preferences/Sensory Perception/Appetite	Altered food tastes with a preference for salty foods. Dietary preference for higher carbohydrates in relation to fat.
Oberg 1981	Salyut 6 (1977–1981)	Sensory Perception (Taste+)/Appetite/Strategies for Maintaining Food Pleasure (Trigeminal)	Canned ham perceived as saltier than usual. Crewmembers experienced cravings for apricot juice, honey, and more spicy and pungent condiments such as mustard and horseradish.
Kerwin and Seddon 2002; Da Silva et al. 2002	Space Shuttle missions (1981–2011)	Strategies for food Enjoyment (Sensory Perception and Human Factors)/Food Preferences	Dietary preference for higher carbohydrates in relation to fat. Some foods tasted bland, leading to the frequent use of condiments like Tabasco sauce. Taste changes and cultural differences in preferred foods sometimes led to reduced appetite or skipped meals.
Douglas et al. 2016; Stuster 2016; NASA 2020	Debrief sessions of ISS crews^2^	Food Preferences/Sensory Perception/Appetite	Food preferences undergo a change from preflight to in‐flight. Altered food preferences with changes in tastes for specific foods and cravings for different types of food while in orbit compared to their preferences on Earth.

*Note*: Subjective experiences were thematically categorized as follows: “Appetite” refers to appetite‐related experiences, including food cravings; “Sensory Perception” covers decreases, increases, or no changes in sensory perception related to flavor (intensity/liking); “Strategies for Maintaining Food Pleasure” involves behavioral strategies related to eating; “Food Preferences” covers any changes in preferences or choices before and during missions; and “Others” includes observations related to chemosensory or oral perception. “Taste+” indicates an increase in intensity perception; “Taste−” indicates a decrease in intensity perception.

Abbreviation: ISS, International Space Station.

#### 3.1.1 Changes in Sensory Perception

Crewmembers from both Soviet and American missions (Baranski et al. 1983; Campton and Benson 1983; Douglas et al. 2016; Perchonok and Bourland 2002; Rambaut et al. 1977) reported changes in taste perception during space missions, often describing food as bland or unappetizing. Notably, the Salyut 6 crew reported an unusually salty perception of canned ham (Oberg 1981). This observation might be attributed to nasal congestion due to fluid shifts in microgravity, which reduces the complexity of flavor profiles (Flores 2008), leaving only the salty taste of the meat more pronounced. Such findings indicate that space conditions may amplify or reduce specific flavor elements, which could vary based on individual taste sensitivity (Dias et al. 2013; Hayes et al. 2010; Pilic et al. 2020). This emphasizes the need for personalized food approaches. Based on the evidence available, fluid shifts associated with microgravity likely lead to nasal congestion, which dulls sensory perception during short‐term missions (Baranski et al. 1983; Smith et al. 2021). However, longer missions may involve complex adjustments to fluid shifts, suggesting that nasal congestion alone may not fully explain the reduced flavor perception experienced over time (Smith et al. 2021; Taylor et al. 2019; Taylor et al. 2020). Furthermore, research on microgravity simulations indicates potential adaptation over time (Antonutto and Di Prampero 2003; Baker et al. 2019; Iwase et al. 2020), though individual variability in fluid adjustment necessitates further study to optimize personalized food presentation and palatability in space environments (Costa et al. 2021). These individual differences warrant further investigation to understand how flavor perception may vary across space travelers.

Based on the author's evaluation of the literature, several strategies could be implemented to address these challenges. Such strategies include personalized meal planning, informed by preflight taste sensitivity assessments, which may help align food offerings with individual sensory preferences, thereby improving intake. In flight, the use of adjustable seasoning systems and encapsulated flavor compounds engineered to release retronasally during mastication could further enhance flavor perception when olfactory input is diminished. These chemical strategies can be complemented by physical and visual enhancements, such as diverse textures and appealing color contrasts, all of which may counteract the monotony and sensory blunting associated with microgravity. Additionally, authors recommend the incorporation of aroma‐releasing compounds, and culturally familiar or emotionally comforting foods may help stimulate appetite and contribute to psychological well‐being. Together, these approaches could mitigate sensory blunting during flight, improve food acceptability, and support both nutritional intake and psychological well‐being on long‐duration missions.

#### 3.1.2 Behavioral Strategies to Enhance Food Enjoyment—Trigeminal Sensations

To counteract reduced flavor perception, space travelers employ behavioral strategies. For example, crewmembers aboard the Skylab and Salyut 6 missions added spices and pungent condiments like mustard and horseradish to their meals (Johnston and Dietlein 1977; Kerwin and Seddon 2002; Oberg 1981; Rambaut et al. 1977). These additions likely stimulate trigeminal sensations, compensating for sensory dullness in space and highlighting the importance of incorporating strong flavors in space menus to support food enjoyment (Brand 2006). While pungent condiments were added to reflect a compensatory response to the diminished flavor perception, it remains unclear whether trigeminal compounds are perceived differently in space. More recent studies suggest that trigeminal‐stimulating compounds, such as the burning sensation caused by small amounts of capsaicin, can influence the perceived thickness of soups regardless of habitual chili pepper intake (Lyu et al. 2021). Designing foods with optimized levels of pungency or astringency could serve as a functional sensory cue, maintaining interest and variety in repetitive menus (He et al. 2023; Pires et al. 2020; Reinbach et al. 2010). Incorporating such stimuli at controlled levels could enhance perceptual contrast without causing discomfort, thereby sustaining sensory interest and palatability, especially in repetitive menus. Personalized condiment kits and sensory trials conducted in space analogs and simulations may further refine these interventions, enabling more tailored and effective flavor delivery systems. Together, understanding how trigeminal simulating compounds are perceived in microgravity could provide strategies for improving the texture and palatability of food in space.

#### 3.1.3 Appetite and Cravings

Space missions also alter appetite and cravings. For instance, Vostok 6 (1963) crews reported a decreased appetite for sweet and an increased preference for pungent foods (Oberg 1981). Interestingly, shifts in preferences particularly cravings for salty foods over sweet ones while in orbit were also observed (Nicogossian 1977; Schroeder and Tuttle 1992). This change may be due to the predominance of sweet foods in astronaut diets to sustain energy, which is critical for maintaining energy balance during space missions (Casaburri and Gardner 1999). On the other hand, salty foods may be craved more due to a general reduction of sodium intake during space missions for health reasons (Lane et al. 2013). Similar findings were observed in ISS crews, who reported cravings for different foods while in space compared to their preferences on Earth (Douglas et al. 2016).

#### 3.1.4 Key Insights and Recommendations

Based on our evaluation of the literature, astronauts consistently report diminished flavor perception during spaceflight, with food often described as bland and preferences shifting toward stronger, more trigeminally stimulating flavors. While fluid shifts and nasal congestion are likely contributors to these changes, it remains unclear whether trigeminal sensations are genuinely heightened in microgravity compared to Earth, or whether such preferences reflect compensatory behavior. In addition, the properties of space food and consumption methods, such as packaging, which often limit aroma release, may also reflect the changes in the flavor perception in space. Despite these observations, there has been little controlled research isolating these factors or quantifying the degree of sensory dullness. We recommend that future studies should incorporate targeted psychophysical testing using portable, space‐compatible methods. These tools should first be validated in appropriate ground‐based analogs, such as microgravity analogs, isolated and confined environments, or digital simulations, where the constraints of space (e.g., limited mobility, altered posture, congestion) can be partially replicated. Examples of potentially suitable methods (depending on study objectives) include Sniffin’ Sticks, filter paper taste strips, and blister‐packed solutions. Moreover, food design strategies, including the integration of pungent compounds or flavor delivery systems optimized for in‐mouth release, should be explored to mitigate flavor dullness and maintain sensory appeal during long‐duration missions. It is important to acknowledge that the feasibility of existing testing methods and food systems in actual microgravity remains unknown, and their suitability must be verified through future testing in spaceflight or validated settings.

### 3.2 Flavor Perception During Spaceflights—Quantitative Sensory Experiments

Table 3 summarizes the findings from studies examining flavor perception changes during spaceflights. To date, only four known sensory functionality experiments (published between 1975 and 1985) have been conducted with space travelers as participants (Baranski et al. 1979; Baranski et al. 1983; Heidelbaugh et al. 1975; Watt et al. 1985). These limited studies underscore the scarcity of systematic sensory research in space, as no additional published studies beyond these have specifically focused on flavor perception in space missions (Olabi et al. 2002).

**TABLE 3:** Spaceflight flavor perception experiments with quantitative data.

Author (year)	Space mission	Sense(s) assessed	Method	Findings
Heidelbaugh et al. 1975	Skylab 4 (1973–1974)	Taste/smell	*n* = 3; detection and recognition thresholds/smell identification; slips of paper impregnated with the four basic tastes (sweet, sour, salty, bitter) and two flavors (orange, onion) at five different concentration levels; range of odorants (lemon, orange, onion, pepper, chicken, wintergreen, chocolate, cherry, spearmint, cinnamon) and a blank control were assessed 10 days preflight, mid‐flight, and 12 days postflight.	Taste (perception decreases for select taste/flavor)/smell (no differences)
Baranski et al. 1979	Soyuz (prior to 30) (1967–1978)	Taste	*n* = unreported; electrogustometry (anodal)^a^	Taste (conflicting direction of results across participants)
Baranski et al. 1983	Soyuz 30–31 (1978)	Taste	*n* = 2; each for Soyuz 30 and Soyuz 31; electrogustometry (anodal; taste detection threshold) assessed preflight, mid‐flight, and postflight before and after mealtimes on different areas of the tongue.	Taste (conflicting direction of results across participants)
Watt et al. 1985	STS‐41G (1984)	Taste/smell	*n* = 2; taste solutions (recognition thresholds)/smell (identification; all/none); prototypical taste solutions with various concentrations were prepared (sweet, sour, salty, bitter) and flavored solutions (lemon, mint, vanilla, blank control) were assessed preflight, mid‐flight, landing day, and postflight.	Taste (no differences)/smell (no differences)

*Note*: Table adapted from Olabi et al. (2002).

^a^ Electrogustometry was used to measure taste detection thresholds by applying a direct current of increasing intensity to the tongue at different areas. The point at which the subject reported a slight sensation was considered indicative of the taste detection threshold (Tomita and Ikeda 2002).

Importantly, these experiments primarily assessed basic sensory functionalities, such as detection and recognition thresholds, rather than affective responses (e.g., hedonic ratings), which limits our understanding of astronauts’ subjective food enjoyment. This focus aligns with the initial objectives of these studies, which aimed to determine whether taste and smell perception changed before, during, and after spaceflight. Notably, three out of four experiments provided more details in their experiments; however, details from the Polish Soyuz missions (Baranski et al. 1979) remain unclear, limiting the interpretation of these results.

#### 3.2.1 Variation in Psychophysical Methods and the Challenges of Consistency

One key difference between the experiments is the range of psychophysical methods used to assess taste thresholds, with each method offering distinct strengths and weaknesses. For instance, taste thresholds refer to detection thresholds (the lowest concentration at which a stimulus can be detected) and recognition thresholds (the concentration at which the taste quality of the sample becomes apparent) (Amerine et al. 2013; Keast and Roper 2007; Low et al. 2017; Snyder et al. 2006). The Soyuz missions employed electrogustometry (Baranski et al. 1979), Skylab 4 used flavor paper strips (Heidelbaugh et al. 1975), and Space Shuttle Mission STS‐41‐G employed taste solutions (Watt et al. 1985). While these variations were likely due to adaptations required for spaceflight, they also complicate direct comparisons of findings and may have contributed to inconsistent results, as each method inherently differs in sensitivity, reliability, and dimensions measured (Lawless and Heymann 2010; Meilgaard et al. 1999).

A particular issue in some studies, such as STS‐41‐G, was a lack of clarity in their methodology for determining taste recognition thresholds, raising concerns about reliability (Olabi et al. 2002). For example, details on the concentration intervals used to assess taste recognition were omitted. The Soyuz missions’ choice of electrogustometry likely aimed to circumvent fluid (taste solution) behavior issues in weightlessness, but this method may also capture trigeminal responses, contributing to variability among individuals due to the diverse sensitivity of receptors (Bartoshuk and Snyder 2016; Olabi et al. 2002; Pavlidis et al. 2021). More recent studies on individual variation in thermal sensations using rods have also found large differences across individuals due to physiological differences in taste receptors and trigeminal nerves (Botha et al. 2021), which may partly explain the inconsistencies in earlier spaceflight studies.

Importantly, a key commonality across these four experiments is the consistent finding that space conditions do not appear to affect smell functionality. However, the data on taste perception changes are conflicting, with some studies indicating changes in taste sensitivity, while others do not. These inconsistencies may be attributed to the large variability among individuals, as previously observed in Earth‐based sensory trials (Cicerale et al. 2012; De Toffoli et al. 2019; Hayes and Duffy 2007; Kato and Roth 2012; Lim et al. 2008; Low et al. 2016, 2017; Mora et al. 2018; Samant and Seo 2019; Webb et al. 2015), since psychophysical studies typically include a minimum of 30 participants to achieve reliable results (Fields 2013). In cases with fewer participants, studies often involve multiple rigorous testing sessions to ensure repeatability (Keast et al. 2003), which is understandably difficult to do during space missions.

Additionally, advances in taste and smell psychophysics since the 1980s—including improved scaling for taste intensity (Bartoshuk and Snyder 2016; Green et al. 1996) and reliable odor discriminatory tests, such as “Sniffin’ Sticks,” with high test–retest reliability scores of 0.7–0.9 (Hummel et al. 1997; Oleszkiewicz et al. 2019)—highlight limitations in these early spaceflight experiments. Standardizing experimental methods in future space missions would allow data from multiple missions to be compiled and analyzed collectively, accounting for individual variability. Taylor and colleagues (2020) also emphasized the need for standardized, easy‐to‐use methods in future spaceflight studies.

Given these challenges, the authors recommend that future studies should adopt standardized psychophysical protocols that are both spaceflight compatible and capable of producing reliable, comparable data. Based on our evaluation of prior studies, solid‐phase taste delivery systems, such as filter paper taste strips or compressed tablets with predefined concentrations, would offer a practical solution. This could eliminate the need for fluid handling while enabling systematic and replicable threshold assessments. Alternatively, taste solutions may also be suitable, provided they are in secure, single‐use modes such as sealed blister packs or pouches that allow controlled release and prevent the risks associated with free‐floating liquids in microgravity. The authors emphasize that the choice of delivery method should balance methodological rigor with operational feasibility to improve the consistency and comparability of sensory data collected in space.

#### 3.2.2 Challenges in Current Methods and Future Research Directions

A notable limitation in most spaceflight sensory experiments is their failure to assess perception at suprathreshold levels, which are more representative of real‐world food experiences (Bartoshuk and Snyder 2016; Keast and Roper 2007). For example, while detection sensitivity is important, studies have shown that taste function measures are independent and can vary significantly across individuals (Low et al. 2016, 2017; Webb et al. 2015). These variations have implications for astronauts’ dietary intake and food choices, as individuals with different taste sensitivities may have differing nutritional needs and food preferences (Low et al. 2016, 2018; Newman et al. 2016).

Although initial space missions did not report significant changes in taste and smell sensitivity, anecdotal reports from astronauts describe a diminished appetite potentially linked to altered taste and smell sensations (Heer et al. 2007). Furthermore, on Earth studies, there is emerging evidence suggesting a coordinated bodily response to nutrients in food between the oral taste receptors in the oral cavity with the receptors located throughout the gastrointestinal tract (Heini et al. 1998; Little and Feinle‐Bisset 2010;Newman et al. 2013). This highlights a need for simplified, yet realistic, sensory tests in future missions—such as those outlined in the International Standards Organisation (ISO) Method for Investigating Sensitivity to Taste (ISO 1991) or Webb et al.’s (2015) adapted suprathreshold procedure—that could better align studies conducted on Earth with sensory data from space missions.

An additional factor to consider in future research is the effect of mission length and the intervals between data collection points on sensory changes. For example, Skylab 4 missions lasted 84 days, observed a decline in taste sensitivity over time, particularly for certain flavors (Heidelbaugh et al. 1975), whereas the STS‐41‐G shuttle mission, which lasted only 8 days, showed no significant differences in taste perception (Watt et al. 1985). These differences may indicate that duration and compound specificity could play roles in sensory alterations, potentially influenced by stress, space sickness, radiation exposure, or sensory deprivation in confined environments (Olabi et al. 2002; Taylor et al. 2020). A recent Virtual Reality (VR) study simulating a confined space environment similarly reported a large variation in odor intensity perception, suggesting that environmental confinement itself may influence chemosensory perception (discussed in Section 4) (Loke, Chand, et al. 2024).

#### 3.2.3 Key Insights and Recommendations

Despite decades of spaceflight, no sensory experiments have been conducted aboard the ISS, and existing studies remain methodologically limited. In our review, most studies focused on threshold type (detection, recognition) methods, often lacking consistent methodologies and affective measures to fully understand interactions with food enjoyment. Small sample sizes, high interindividual variability in sensory sensitivity, and inconsistent testing protocols underscore the need for standardized, scalable psychophysical methods. Although the small sample sizes in these studies limit the broader generalizability of findings, the authors acknowledge that such constraints are inherent in space‐related research, where participant numbers are naturally limited to a highly selective and constrained astronaut population. Further, methods should be tested across ground‐based analogs to ensure robustness under space‐relevant stressors. For instance, food samples may be administered through sealed delivery systems compatible with the ISS environment. Sensory responses could be assessed using either hedonic methods to evaluate liking and acceptability or descriptive methods to characterize flavor intensity and quality. Depending on the research aim, appropriate scale formats such as hedonic scales for liking or magnitude estimation scales for intensity should be selected. Data collection should be optimized using portable or digital entry systems that minimize equipment burden and allow standardized input in confined or resource‐limited environments. We also observe that mission duration and compound‐specific factors likely influence sensory responses, reinforcing the importance of tailoring sensory evaluation methods to different spaceflight stressors. For example, studies may stratify participants based on pre‐flight sensory sensitivity or use crossover designs to track intraindividual shifts across mission phases. Moreover, most studies capture only single time‐point data, limiting insight into the progressive nature of sensory adaptation over the course of a mission. While early reports suggest that fluid shifts and nasal congestion contribute to altered taste and smell, we propose that long‐duration missions may introduce additional physiological and psychological factors that further affect flavor perception. In particular, the distinction between early‐phase changes linked to fluid shifts and later‐phase adaptations associated with sustained microgravity remains poorly understood. We recommend that future research prioritize the development of standardized psychophysical testing protocols that incorporate suprathreshold intensity measures and repeated‐measures designs, alongside the use of analog and simulation‐based missions (as discussed below), to enable meaningful comparisons across different mission types and experimental contexts. These analog environments should serve as iterative testing grounds where methodological adjustments, such as delivery method, exposure timing, and participant comfort, are refined before potential implementation in true microgravity. However, we emphasize that this review aims to provide methodological direction rather than fixed testing protocols. The precise combination of tools, measures, and schedules will depend on specific research aims, hardware constraints, and the operational feasibility in spaceflight. These approaches will better capture mission‐relevant sensory changes and support the development of effective flavor‐based countermeasures to enhance astronaut nutrition and well‐being.

## 4 Results and Discussions—Space Condition Analogs and Simulation Studies and Applicability for Studying Flavor Perception

This section identifies and assesses analogs and simulations for studying flavor perception, focusing on understanding sensory adaptations in space‐like conditions. In this review, “space analogs” refer to Earth‐based environments that mimic specific aspects of space conditions, including microgravity, isolation/confinement, and low‐gravity environments (NASA 2023). “Space‐related simulations,” in contrast, involve highly controlled setups replicating specific aspects of space conditions (NASA 2024). These analogs and simulations are valuable because they allow researchers to isolate the impact of isolation, confinement, and microgravity on sensory perception, which is crucial for understanding flavor perception in space. Gaining this understanding can inform the design of interventions to maintain sensory satisfaction, optimize serving and feeding systems, and develop food formulations that compensate for altered perception and preferences, ultimately improving astronaut well‐being and dietary adherence during missions.

A total of 29 articles were identified as space analogs (*n* = 23) and simulation methods (*n* = 6) for sensory perception‐related studies. These methods are summarized in Supporting Information B, which includes their suitability for flavor perception studies. The applicability was judged based on participants’ ability to taste and rate sensory stimuli (e.g., tastants and odors).

### 4.1 Overview of Flavor Perception Studies—Space Condition Analogs and Simulation Studies (*n* = 29)

**FIGURE 3:**
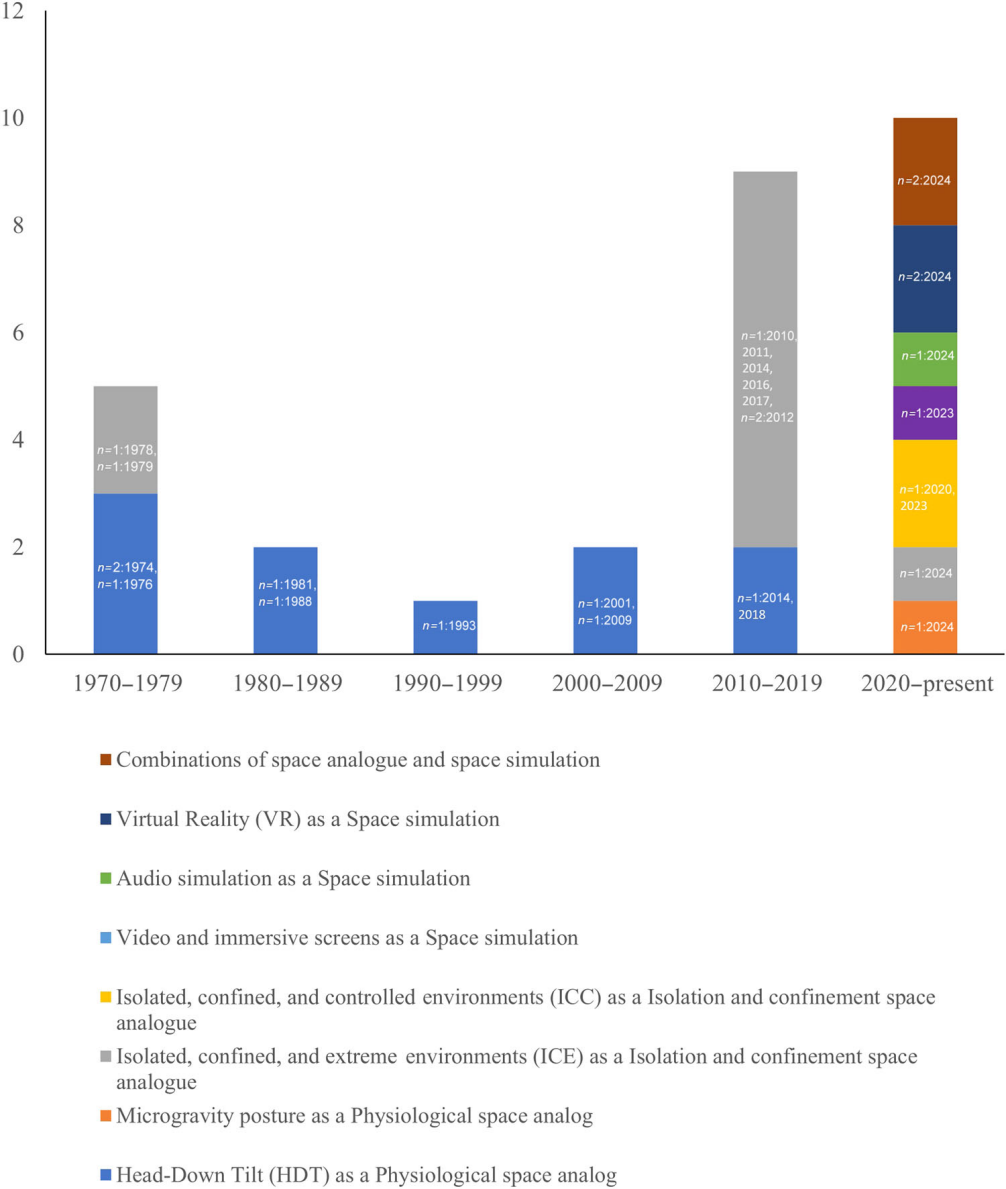
Space analogs and space simulation methods used in sensory research within 28 studies. The *y*‐axis shows the frequency of studies, and the *x*‐axis represents space analog and space simulation types. Stacks highlight specific methods such as head down tilt (HDT) and microgravity posture as microgravity analogs; isolated, confined, and extreme (ICE) and isolated, confined, and controlled (ICC) environments as isolated and confinement analogs; and video and Virtual Reality (VR) simulations as space simulations. The number of articles and publication years are also included.

The identified studies were published between 1974 and 2024, with an increase in research activity from 2010 to 2024 (Figure 3). This corresponds directly with the construction (2000–2010) and operation (2010–current) periods of the ISS, during which time there has been a permanent human presence in space. These studies were conducted in the United States (*n* = 9; 1988–2020), Russia (*n* = 8; 1974–2023), Australia (*n* = 5; 2023–2024), and other regions such as Canada (*n* = 2; 2023–2024), Antarctica (*n* = 3; 1978–2024), Japan (*n* = 1; 1993), and Germany (*n* = 1; 2014). Several studies (*n* = 10) are associated with space agencies, such as NASA and the European Space Agency (ESA). The authors have cross‐checked references and translated several articles, but any papers that could not be translated into English were not considered for this review. It was also noted that there was a significant increase in published papers from Australia focusing on flavor perception in food using digital simulation methods in 2024 (*n* = 6; 2023–2024).

### 4.2 Space Condition Analogs and Relative Merits for Studying Flavor Perception

Of the 23 identified space analog studies, two types were identified: isolation and confinement analogs (*n* = 12) and physiological analogs that mimic physiological adaptation process to microgravity (*n* = 11). Additionally, space agency‐related analogs were reviewed and classified, with details summarized in Tables S2 and S3.

#### 4.2.1 Isolation and Confinement Analogs/Analog Missions

Isolation and confinement analogs are generally classified into:

1. Isolated, confined, and extreme (ICE) environments: Remote and extreme locations focusing on field research and training exercises (Cromwell et al. 2021).
2. Isolated, confined, and controlled (ICC) environments: Controlled conditions focusing on controlled research studies to examine the effects of isolation (Cromwell et al. 2021).

Table S2 summarizes details of each analog's environment and study areas. It is noted that these analogs have not focused specifically on flavor perception, but they could potentially be used for future sensory studies. Examples of methods include Arctic and Antarctic missions, Mars Desert Research Station (MDRS), and facilities such as HERA (NASA), NEK (IBMP, Russia), HESITA (DLR, Germany), and CHAPEA (NASA).

##### 4.2.1.1 Flavor Perception in ICE Environments (*n* = 10)

Table 4 summarizes the findings from studies on flavor perception and sensory changes in ICE environments. While flavor‐specific studies remain limited, studies have highlighted that environmental stressors, psychological adaptation, and the effects of isolation can influence chemosensory perceptions, appetite, and enjoyment of food. For instance, behavioral data from these missions primarily address the effects of extended confinement and sensory monotony (Basner et al. 2014; Bell et al. 2019; Kahn and Leon 1994; Kanas et al. 2011; Le Roy et al. 2023; Marques‐Quinteiro et al. 2023; Palinkas and Suedfeld 2008; Tafforin et al. 2015; Van Fossen et al. 2021). Such sensory deprivation may occur due to physical constraints or limited food variety (Bachman et al. 2012; Stuster 2016). For example, astronauts reported that food lacks the same taste as it does on Earth (“it's tough to get enough food in the belly because it doesn't taste good as it does at home” [Stuster 2016]). While such data are valuable, they underscore a recurring gap: the limited quantitative assessment of sensory thresholds and taste quality changes over time to clarify the extent and mechanisms of these perceptual changes. Bridging this gap will require integrating validated sensory metrics into mission designs, even within resource‐constrained analog environments.

**TABLE 4:** Flavor perception experiments in isolated, confined, and extreme (ICE) analogs.

Author (year)	Analog mission	Sense(s) assessed	Method	Findings
Barabasz and Gregson 1979	Antarctic wintering over—Scott Base, Antarctica (December 1976 to October 1977)	Smell	*n* = 9; physiological responses (EEG and SC responses) to real and suggested odorants were measured in pre‐ and post‐wintering over; odorants presented on sterile glass rods held near the nose; odorants: acetone, ammonia, eugenol, isoamyl acetate, and propanol; turpentine was assessed 8 weeks after arrival and 10 months later	Increased olfactory sensitivity to real odors and suggestibility to suggested odors in post‐isolation.
Gregson 1978	Antarctic wintering over—Scott Base, Antarctica (December 1976 to October 1977)	Taste	*n* = 9; 5 mL of four glucose solutions (0.35%, 0.70%, 1.40%, 2.80%) and four citric acid solutions (0.0012%, 0.0025%, 0.0050%, 0.0100%); intensity ratings were assessed on a 0–10 scale (from *Very faint* to *Extreme*) and qualitative identification of the taste “sweet,” “salty,” “sour/acidic,” “bitter,” or “no taste,” or other spontaneous comments such as “orange drink” or “disprin” before and after the wintering‐over period	Taste identification and intensity perception remained stable, with no significant deterioration in wintering over while slight improvement in both taste identification and intensity perception for glucose in post‐wintering. Citric acid identification with frequent confusions between “sour” and “bitter/salty,” both during and after isolation. Intensity ratings increased with concentration.
Hunter et al. 2011	Mars Desert Research Station (MDRS) (2009–2010)	Taste	*n* = 61; sensory evaluations (appearance, aroma, flavor) and the assessments of food monotony, satisfaction, and intake for “instant” meals and cooking custom‐prepared meals from shelf stable ingredients; documented social dynamics during meal preparation and consumption	Custom‐prepared meals were rated higher than instant meals. Satisfaction and food intake were greater on cooking days, while instant meals contributed to monotony.
Rai and Kaur 2012	Mars Desert Research Station (MDRS)	Taste	*n* = 12; two sessions of 1 h—mental workload and physical workload; taste sensitivity tests for quinine sulphate (bitter), citric acid (sour), and sucrose (sweet) before and after mental and physical tasks; salivary biomarkers measured before and after tasks; psychological mood states are assessed using POMS and CST.	Taste intensity and duration of aftertaste for all three tastes were significantly reduced after both physical and mental stress. Physical workload induced more pronounced effects on taste than mental workload. A correlation was found between taste perception changes and elevated salivary cortisol levels and CST scores.

Abbreviations: CST, current stress test; EEG, electroencephalogram; POMS, Profile of Mood States; SC, skin conductance.

###### 4.2.1.1.1 MDRS Studies on Sensory Experiences

During the Mars 500 mission, prolonged confinement and a monotonous diet were shown to reduce appetite and affect psychological well‐being (Šolcová et al. 2016; Ushakov et al. 2014). Further, Hunter et al. (2011) found that instant meals contributed to food monotony, while custom‐prepared meals with diverse flavors, appearances, and aromas increased satisfaction and food intake on designated cooking days. The effect of varied flavors on alleviating sensory monotony could be a critical area for future studies. Similarly, Rai and Kaur (2012) observed that physical and mental workloads decreased taste sensitivity (sweet taste), particularly during physically demanding tasks, pointing to a gap in research around complex flavors and psychological resilience. In comparison to other taste qualities, the sweet taste quality consistently yielded the highest identification rates throughout the trial. Notably, a significant decrease in cumulative salty taste scores was observed over the course of the Antarctic stay, returning to baseline levels at the end of the trial. These findings suggest that certain taste qualities may be more sensitive to isolation and stress than others. Future research should aim to systematically investigate how different taste qualities are affected under such conditions, using standardized stimuli and blinded protocols to ensure reliable and comparable results.

###### 4.2.1.1.2 Antarctic Winter‐Over Studies on Sensory Experiences

Antarctic winter‐over missions provide additional evidence suggesting that altered chemosensory experience is more attributable to prolonged stays in extreme environments than to sensory acuity loss (Barabasz and Gregson 1979; Gregson 1978). Although changes in taste perception over extended stays remain uncertain, studies have found stable sweet and sour taste perception during winter‐over, with slight improvements in taste sensitivity post‐isolation (Gregson 1978). These findings contrast with subjective observations from Mars 500, where preference for sweets declined (Agureev et al. 2017). Another recent 1‐year study at Concordia Station in Antarctica (*n* = 19) observed a general decline in olfactory function and an increase in hyposmia over the course of the expedition. Gustatory function remained largely stable, though sensitivity to salty tastes diminished during isolation. These findings suggest that the combined effects of isolation, confinement, and hypoxia may contribute to progressive sensory changes, potentially influencing dietary habits, energy intake, and body weight (Klos et al. 2024).

##### 4.2.1.2 Flavor Perception in ICC Environments (*n* = 2)

While flavor‐specific studies remained very limited in the ICC environment (*n* = 1; Table 5), these missions serve as a platform for controlled research studies, examining the impact of isolation and mission‐related stress on the crew and the behavioral effects on humans like those experienced in space. Similar to the findings in the MDRS study (Hunter et al. 2011), NASA's HERA facility reported that repetitive meal consumption led to reduced satisfaction and increased stress (Sirmons et al. 2020). Notably, allowing participants to cook or have custom‐prepared meals enhanced morale and overall satisfaction, underscoring the role of food variety and enjoyment in sustaining sensory engagement (Hunter et al. 2011). This highlights that flavor variety and customization may help mitigate menu fatigue and reduce the risk of sensory decline over time. Analog studies could benefit from incorporating rotational meal plans or personalized menus to examine whether increased sensory novelty can improve satisfaction, dietary adherence, and overall well‐being in prolonged confinement settings. However, the absence of diverse flavor profiles within the mission's menu limits the scope of insights into adapting to complex flavors under confinement. Furthermore, the SIRIUS‐21 analog mission examined how repeated food consumption affects acceptability, preference, and menu fatigue over an 8‐month simulated spaceflight. Preliminary results indicate variability in food acceptability over time, with trends suggesting potential decreases in ratings and preferences for certain items, highlighting the need to optimize food systems for long‐duration missions (Douglas et al. 2023).

**TABLE 5:** Flavor perception experiments in isolated, confined, and controlled (ICC) analogs.

Author (year)	Analog mission	Sense(s) assessed	Method	Findings
Sirmons et al. 2020	NASA HERA 30‐day mission (January 2016 to October 2016)	Taste	*n* = 16; MRBs: two sweet flavors (banana nut, orange cranberry) and two savory flavors (BBQ Nut, Jalapeño Nut); subjects consumed MRB daily for 15 days, then intermittently for another 15 days. SMF was provided on non‐MRB days. Sensory evaluations (appearance, aroma, flavor, texture) were rated daily using a 9‐point hedonic scale. Appetite, satiety, and cravings were measured pre‐ and post‐breakfast daily. Behavioral health measures (POMS‐SF, PANAS) were assessed at the end of each day, and body weight was measured daily.^a^	Sweet MRBs were rated higher than savory ones, with BBQ Nut and Jalapeño Nut receiving the lowest ratings. Daily MRB consumption led to less satisfaction, increased stress, and monotony and caloric deficits.
Douglas et al. 2023	SIRIUS‐21 for 8 months	Taste	*n* = 5; weekly food acceptability questionnaires using a 9‐point hedonic scale assessed foods consumed at three meals every 6 days, capturing in situ scoring of repeat consumption. Open‐ended feedback on food context and attributes was collected alongside monthly surveys to characterize the food system, any changes, and overall experience.	Preliminary results show variability in food acceptability, with trends indicating potential decreases over time, suggesting menu fatigue and the importance of optimizing food systems for long‐duration missions.

Abbreviations: BBQ, barbeque; MRB, meal replacement bars; SMF, standard menu foods.

^a^ Behavioral health measures included the Profile of Mood States—Short Form (POMS‐SF) and the Positive and Negative Affect Schedule (PANAS).

##### 4.2.1.3 Key Insights and Recommendations: Flavor Perception Research in Analog Missions

Flavor perception research in space analog environments remains significantly constrained by several methodological and conceptual limitations. Based on the authors’ evaluation of the current literature, a primary challenge is the limited focus on sensory studies within these settings. While gustatory changes have been occasionally explored, flavor perception is often treated as a secondary consideration. As a result, the available research lacks both depth and consistency.

In the authors’ view, analog environments such as Antarctic overwintering missions and the MDRS offer valuable and currently underutilized opportunities to examine the effects of isolation on human perception. Expanding sensory studies in these settings could address existing gaps and provide more robust insights into how isolation impacts flavor perception.

A further critical limitation is the insufficient consideration of individual variability. Baseline taste sensitivity, dietary habits, and personal food preferences can significantly impact chemosensory responses. However, many analog studies do not account for these factors, resulting in wide variability and reduced reliability of conclusions. The authors recommend that future studies stratify participants based on pre‐assessed chemosensory profiles or include repeated within‐subject designs to better understand intra‐ and interindividual differences.

Another key limitation is the small sample size inherent to analog missions, which restricts statistical power and generalizability. While these studies typically involve limited participants due to logistical constraints, their scientific yield can be improved through repeated planned designs. Such strategies increase statistical validity and allow investigating flavor perception under confinement. Additionally, researchers might analyze existing dietary records or food choice to infer food preferences and intake patterns. Despite these challenges, the authors assert that analog environments hold strong potential for advancing flavor perception research. As demonstrated in studies conducted in Antarctica, MDRS, and NASA's HERA facility, it is feasible to implement structured sensory testing in isolated and confined conditions. These settings offer unique ecological validity for understanding the effects of prolonged confinement, psychological stress, and menu monotony on sensory experience.

#### 4.2.2 Physiological Analogs for Microgravity Simulation (*n* = 11)

Physiological analogs simulate physiological adaptation process as in microgravity but do not fully replicate space conditions (Cromwell et al. 2021; Ferranti et al. 2020; Oluwafemi and Neduncheran 2022). In life sciences, various analogs have been utilized to mimic the effects of microgravity (see Supporting Information B). Table S3 provides a summary of facilities offering physiological analogs. Of note, these are government‐based facilities, and known private sectors are also offering these facilities for research (but no published studies have been found so far). Notably, most recent published studies identified two approaches to simulate small components of microgravity in conducting sensory‐related studies. This includes the microgravity position (simulated body fluid movements that occur during microgravity) and microgravity postures (simulated posture of space travelers during microgravity).

##### 4.2.2.1 Flavor Perception in Physiological Analogs for Microgravity Position (*n* = 10)

Table 6 summarizes flavor perception experiments conducted in physiological analog for microgravity position, designed to simulate the physiological effect of body fluid shift. Generally, bed rest and dry immersion analogs induce microgravity‐like effects on the body (Ferranti et al. 2020; Hargens and Vico 2016; Navasiolava et al. 2011; Tomilovskaya et al. 2019), while parabolic flights provide alternating phases of hypergravity (1.8–2 g) and brief periods of true microgravity (∼20 s) (Ogoh et al. 2018; Shelhamer 2016). To date, flavor perception studies have only been conducted using bed rest as a physiological analog for microgravity position. These experiments were comprehensively reviewed by Olabi et al. (2002), and since then, only four additional published studies (Cromwell et al. 2018; Gonzalez Viejo, Harris, and Fuentes 2024; Loke, Chandrapala, et al. 2024; Low and Loke 2024; Rai 2009) have been identified to investigate the use of these analogs concerning flavor perception. The key findings from these studies are discussed below to evaluate how these analogs have been applied to flavor perception and eating experience research.

**TABLE 6:** Flavor perception experiments in microgravity analogs.

Author (year)	Experimental condition	Sense(s) assessed	Key methodology	Key findings
Kurliandskii et al. 1974	−4° HDT bed rest (hypokinesia) 30 days	Taste	*n* = 15; functional mobility method^a^	Increased taste thresholds for sweet, salt, acidic, and bitter. Decreased taste sensitivity, appetite, and mobilization of taste receptors in the early bed rest period due to hypodynamia
Volozhin et al. 1974	Hypodynamia condition 30 days	Taste	*n* = 15; taste sensitivity using taste threshold and functional mobility method^a^	Decrease in taste sensitivity and decreased mobilization of taste receptors of the tongue
Budylina et al. 1976	+6, −2, and −6 bed rest 30 day	Taste	*n* = unreported; taste sensitivity using functional mobility method^a^	Reduced taste sensitivity and an elevated mobilization of taste receptors of the tongue in +6 and −2, while −6 yielded opposite changes in the thresholds of taste sensitivity and phasic changes in the mobilization of taste receptors.
Yakovleva 1981	−8° HDT bed rest 5 days (AOH)	Taste	*n* = 62; electrogustometry before and after food intake. *n* = 53 (controls) and *n* = 9 were examined for 5 days −8° bed rest; thresholds were measured before (baseline) and after food intake at various intervals (days 1, 3, 5) during the AOH period^b^	Taste thresholds increased significantly during the AOH period but tended to normalize by the fifth day. AOH induces alterations in taste sensitivity, characterized by increased thresholds and changes in the gastrolingual reflex.
Mester et al. 1988	0° (upright), 90° (supine), 135°, 180° (upside down) positions within sagittal plane	Smell	*n* = 16; blood pressure, heart rate, olfactory function, and nasal resistance were tested; olfactory function measured by 10‐item booklet of UPSIT: scratch and sniff test imbedded in 10‐ to 50‐µm microcapsule fixed on brown strips of each booklet page and MCQ with four responses including smell types.	Odor identification scores decreased as a function of the degree of tilt
Kanda et al. 1993	Upright and HDT position	Taste	*n* = unreported; taste sensitivity was measured	No significant change in taste sensitivity or food acceptance between the upright and the HDT conditions
Vickers et al. 2001	−6° HDT bed rest 15 days	Taste and smell	*n* = 6; five tastants (sucrose, sodium chloride, citric acid, quinine, and MSG), one trigeminal stimulus (capsaicin), and two odorant samples (iso‐amyl butyrate and menthone); sample serving: series of 10 dilutions for each substance, with dilution 10 being the highest concentration.	Increment in detection threshold for MSG by one dilution step, while no effect on others. No significant effect of self‐reported congestion on thresholds for any of the flavor compounds.
Rai 2009	−6° HDT bed rest 12 h	Taste and smell	*n* = 10; five tastants (sucrose, sodium chloride, citric acid, quinine, and MSG), one trigeminal stimulus (capsaicin), and two odorant samples (iso‐amyl butyrate and menthone); sensory threshold was measured.	Sensory threshold for MSG increased by 1.5 dilution. Threshold level of capsaicin increased, while sodium chloride decreased.
Enck et al. 2014	−6° HDT bed rest 5 days (with high‐ and low‐sodium diet)	Smell	*n* = 8; Sniffin’ Sticks test (odor threshold, discrimination, and identification); samples: phenyl ethyl alcohol for odor threshold test, 16 pairs of similar odorants for odor discrimination test, and 16 common odorants (e.g., vanilla, peppermint) for odor identification test.	Significantly reduced olfactory sensitivity (increased odor threshold, decreased discrimination). The high‐sodium diet further reduced odor discrimination.
Cromwell et al. 2018	HDT bed rest at NASA Flight Analogs Research Unit 70‐day	Smell	*n* = 16; two groups—a high‐exercise group and a low‐exercise group; assess odorant identification, nasal patency, and food acceptability; UPSIT^c^: 40 scratch‐and‐sniff odorants (both food related and non‐food related); administered twice pre‐HDT, four times during HDT, and twice post‐HDT; Odorant Identification Test: 35 food‐related odorants were encountered 28 times (pre‐HDT, during HDT, and post‐HDT) for orthonasal and retronasal smelling. Odorants included menu items, non‐menu items, and calibrates. Ratings on a VAS for intensity, familiarity, pleasantness, and acceptability; identification from a list of probable, improbable, and exact names.	Significant decline in smell discrimination for the low‐exercise group during HDT; the high‐exercise group showed no significant changes. In post‐HDT recovery, low‐exercise group scores remained lower than pre‐HDT. Variability in odorant identification accuracy, with menu odorants showing both high and low identification rates.

*Note*: Table adapted from Olabi et al. (2002).

^a^ Functional mobility method is a process that determines the physiological ability of the body to adapt by weakening or strengthening system functions through the reduction or increase in the number of active functional units (Sniakin 1971).

^b^ Electrogustometry was used to measure taste detection thresholds by applying a direct current of increasing intensity to the tongue at different areas. The point at which the subject reported a slight sensation was considered indicative of the taste detection threshold (Tomita and Ikeda 2002).

^c^ UPSIT—The University of Pennsylvania Smell Identification Test is a standardized test that measures the individual's ability to detect odors at a suprathreshold level (Doty et al. 1984).

Abbreviations: AOH, antiorthostatic hypokinesia; HDT, head down tilt; MCQ, multiple‐choice questions; MSG, monosodium glutamate; NASA, National Aeronautics and Space Administration; UPSIT, University of Pennsylvania Smell Identification Test; VAS, Visual Analog Scale.

###### 4.2.2.1.1 Head Down Tilt (HDT) Bed Rest Studies on Taste and Smell

Generally, studies reveal diverse methodological approaches in sensory psychophysics. Both Vickers et al. (2001) and Rai (2009) conducted experiments using sensory setups with small dilution steps and repeated measures to examine changes during −6° HDT bed rest periods. Specifically, Vickers et al. (2001) conducted a 15‐day HDT bed rest study with a dilution series for tastants and odorants, covering basic tastes like sucrose, sodium chloride, citric acid, quinine, and monosodium glutamate (MSG), alongside capsaicin (trigeminal stimuli) and odorants (iso‐amyl butyrate, menthone). The same samples were used by Rai (2009) for a shorter study of 12 h measuring thresholds but with unspecific methodologies (see Table 5). Both studies reported an increase in MSG thresholds, indicating a decline in sensitivity to umami, with Rai (2009) additionally noting heightened capsaicin thresholds and reduced sodium thresholds, suggesting variable effects across taste modalities under HDT conditions. While these findings are promising, limitations due to small sample sizes (range from six to 10) and variation in experimental duration present challenges in forming hypotheses. Notably, aligning multiple bed rest studies may enhance sample power and reliability, offering a pathway for investigating sustained fluid shift impacts on flavor.

Interestingly, studies led by Volozhin et al. (1974), Kurliandskii et al. (1974), Yakovleva (1981), and Budylina et al. (1976) focused on the impact of simulated weightlessness on taste receptors on the tongue. These studies revealed varied effects, with Kurliandskii et al. (1974) and Yakovleva (1981) reporting reduced taste sensitivity, supporting the general trend of reduced sensitivity under prolonged bed rest, a pattern also noted in studies of physical inactivity (Bortz II 1984). In contrast, Budylina et al. (1976) observed an increased sensitivity with phased receptor activation in highly antiorthostatic conditions and reduced sensitivity and increased receptor activity in low tilts. Further, Kanda et al. (1993) found no significant difference in taste sensitivity between upright and HDT and consistent food acceptability across gravitational conditions. A more comprehensive review of these findings is available in Olabi et al. (2002)​, which underscores the need for longitudinal studies to determine the persistence of sensory changes under HDT​.

Further evidence of sensory decline under HDT conditions was found by Enck et al. (2014) in a 5‐day −6° HDT bed rest study (*n* = 8), which assessed olfactory function for odor threshold using phenyl ethyl alcohol in a series of dilutions, discrimination, and identification using 16 common odors, such as peppermint and vanilla. Results showed a significant reduction in olfactory sensitivity mainly due to impaired odor discrimination. Notably, a high‐sodium diet further reduced discrimination ability, suggesting an interaction between salt intake and olfactory function under HDT conditions. Moreover, one of the most comprehensive studies so far was a 70‐day NASA HDT bed rest study (*n* = 16), which assessed the influence of exercise on olfactory function, dividing participants into high‐ and low‐exercise groups (Cromwell et al. 2018). The study employed a thorough sensory assessment (Table 5), including a validated scratch‐and‐sniff test with 40 odorants (both food and nonfood), measured pre‐HDT, during HDT, and post‐HDT, alongside an odor identification test involving food‐related smells. Interestingly, the low‐exercise group showed a significant decline in smell identification during the bed rest period, while the high‐exercise group maintained stable olfactory performance. Additionally, odor identification varied, with some food‐related odors being identified more easily than others. The findings could be supported by more recent exercise studies (Gauthier et al. 2023; Gauthier et al. 2020; Namiranian et al. 2024), where physical activity status was positively associated with chemosensory performance, underscoring exercise as a potential countermeasure to sensory decline in prolonged inactivity. This suggests the need for future research into the role of external factors like exercise (particularly the length equivalent in microgravity conditions) in aiding long‐term sensory functionality and flavor sensitivity maintenance during longer term missions.

##### 4.2.2.2 Flavor Perception in Microgravity Chair Studies (*n* = 1; Published Year >2024)

Apart from HDT bed rest, microgravity chairs simulate specific postural conditions associated with space travel in 1g (0°–170°; NASA neutral posture during microgravity), although they lack fluid redistribution effects typically found in HDT. The method does not require specialized equipment as they have been commercialized to backyard/massage chair products that are easily accessible (NASA's spin‐off product of microgravity postures based on Skylab missions) (NASA 1997, 2013). Table 7 summarizes the findings of these studies. Of highlight, Gonzalez Viejo, Harris, and Fuentes (2024) are the first to assess basic tastes, aromas, and mouthfeel using microgravity chairs alone. This study was assessed with 14 trained panelists in three seating positions: normal (90°–270°), semireclined (55°–135°), and “microgravity” postures (0°–170°). Results indicated that tastes were perceived with higher intensity in microgravity postures, while aroma perception decreased, suggesting a modality‐specific response to postural changes. Mouthfeel intensity showed no significant differences as well. These findings may be attributed to chance due to the small sample size.

**TABLE 7:** Flavor perception experiments in microgravity postures.

Author (year)	Experimental condition	Senses assessed	Key methodology	Key findings
Gonzalez Viejo, Harris, and Fuentes 2024	Normal position (90°–270°), followed by a semireclined position (55°–135°) and a microgravity position (0°–170°)	Taste, smell, and mouthfeel	*n* = 14 (trained panelists); tastants: sweet, sour, salty, bitter, and umami; odorants: Cinnamon, roasted almond, rose, banana, orange, and butter; mouthfeel: pungent/hot (chili flakes), refreshing (mint drops), cold (ice), astringent (90% cocoa chocolate), and popping (popping candy); intensity ratings using 15‐cm nonstructured intensity scales; biometric data (heart rate, blood pressure) and emotional responses were recorded.	Lower aroma intensity and higher taste intensity in microgravity position compared to normal position. Reduced heart rate and blood pressure in microgravity in aroma and taste sessions.
Low and Loke 2024	Neutral sitting, NASA neutral posture^a^ (122°–124° posture), and space VR simulation	Odor	*n* = 44; intensity of three odors (vanilla, lemon, almond) and control on a 5‐point Likert scale across three conditions	No significant differences in odor perception between neutral and NASA neutral postures^a^ for odors. Significant differences were observed in vanilla and almond in VR compared to both postures.
Loke, Chandrapala, et al. 2024	Microgravity posture and VR	Odor	*n* = 44; intensity of eight odors (vanilla, almond, lemon, lemongrass, lemon myrtle, eucalyptus, peppermint, and vinegar) and control on a 5‐point Likert scale and emotional dimensions	Higher odor intensity perception in VR for all odors except lemongrass. High odor intensity for almond by high‐sensitivity participants, while high‐intensity perception for vinegar by low‐sensitivity participants in VR.
Gonzalez Viejo, Harris, Tongson, et al. 2024	Normal and simulated microgravity using reclining chairs (positions) with neutral and space‐immersive environments	Taste, aroma, and texture	*n* = 51; samples: six leafy greens—basil, sweet basil, coriander, kale, mixed cos lettuce, and beetroot; affective questions using a 15‐cm nonstructured scale and emotional responses using CATA; biometric responses were collected through videos; univariate and multivariate analyses were conducted to assess self‐reported and biometric consumer sensory analysis.	A significant difference in the room × position interaction for head movements; significant differences in the sample × position interaction for all liking attributes; and nonsignificant differences in the samples × environment interaction.

Abbreviations: CATA, check all that apply; VR, Virtual Reality.

^a^ NASA neutral posture mimics “microgravity” posture using a commercial “zero‐gravity” outdoor chair set at 122°–124° (Low and Loke 2024).

While trained panelists provided a controlled starting point for sensory testing, it may limit the generalizability of the results, as astronauts and general populations are not typically trained sensory assessors known for their heightened sensory acuity. Additionally, the study's inclusion of trained panelists’ emotional and physiological responses introduces complexity. Such measures are less common in trained panel type study, as affective evaluations—being subjective and based on personal differences—may be influenced by biases from training and repeated exposure to stimuli (Lawless and Heymann 2010). Although this study provides valuable insights into posture‐related sensory changes, its applicability to space missions requires careful consideration. Although trained panelists’ procedural familiarity minimizes extraneous variability, they are more reliable for detecting posture effects. However, the emotional and physiological responses may not reflect those of astronauts as they typically lack the trained sensory sensitivity of panelists, limiting the relevance of such measures to real‐world space environments. Nonetheless, the exploration of posture‐related sensory variations provides a foundation for understanding how microgravity postures could impact sensory perception and inform the design of foods and environments in follow‐up studies by the team.

In another recent study, Loke, Chandrapala, et al. (2024) examined a larger sample size of 44 participants using a similar microgravity chair setup. This study focused on orthonasal aroma intensity perception across neutral and microgravity postures, revealing that differences between postures may be aroma specific. However, the selection of odors was limited, but the data showed the potential of the microgravity posture chair to improve space‐like presence when combined with other analogs or simulation methods (discussed in Section 4.3). Overall, a key limitation of these studies is the lack of rigor in classical psychophysical study designs, such as incorporating multiple concentration levels and repeated measures. Despite this, the current method is promising for future research applications that mimic short‐term effects.

###### 4.2.2.2.1 Further Insights from Body Tilt Studies

Mester et al. (1988) found that increased body tilt within the sagittal plane—0° (upright), 90° (supine), 135°, and 180° (upside down)—impaired odor identification performance, potentially due to tilt‐induced changes in cephalic circulation. However, nasal resistance measurements remained unchanged, possibly due to the limitations of the measurement techniques used. Later studies by Lundström et al. (2006, 2007) confirmed that body position affects olfactory sensitivity at peri threshold levels, suggesting that cognitive factors, rather than blood flow changes, may play a more significant role. In the authors’ view, while the role of cognitive factors in peri threshold olfactory detection is plausible given the influence of attention and perception, the limited physiological evidence suggests that the contribution of circulatory changes cannot yet be definitively excluded particularly under conditions involving altered posture or gravity. Hort et al. (2008) observed no significant impact of body position on retronasal flavor discrimination, yet emphasized the need for further research with larger sample sizes to better understand the influence of posture on different aspects of flavor perception. These findings collectively provide a foundation for exploring how body posture may alter flavor perception, potentially through cognitive and sensory mechanisms that interact with environmental and physiological factors.

##### 4.2.2.3 Key Insights and Recommendations: Flavor Perception Research in Physiological Analogs

Physiological analogs, such as HDT studies, have been primarily employed to investigate general physiological and psychological responses to space‐like conditions. In the author's view, flavor perception has typically remained a secondary focus, resulting in limited advancement of sensory‐specific research relevant to long‐duration spaceflight. A number of methodological limitations further constrain the scope of current research. One key example is the reliance on electrogustometry, which is incapable of detecting nonionic taste qualities such as sweet and bitter. Moreover, by isolating taste from olfaction, it fails to capture the multisensory nature of flavor perception. Additionally, the method also does not account for the microgravity‐specific conditions such as nasal congestion, which can significantly affect overall flavor experience. Instead, authors recommend employing simplified tools such as filter paper strips or sealed liquid samples or Sniffin’ Sticks that preserve quality and safety under posture‐induced fluid shift conditions. The selection of these tools should be guided by the study's objective. For instance, if the goal is to assess sensory sensitivity, filter paper strips may be more appropriate for dry compounds, whereas blister‐packed liquids may be required for soluble tastants. It is also essential to ensure that packaging maintains sample integrity throughout testing. In the case of olfactory testing, practical issues such as how to administer tools like Sniffin’ Sticks under altered posture or in microgravity (e.g., difficulty inserting into the nasal cavity safely and consistently) must be addressed. Importantly, if these tools are to be used in actual microgravity environments, comparable methods should be employed across relevant analog settings to ensure consistency and facilitate meaningful comparisons across conditions. This highlights the need for a broader planning framework that aligns testing procedures across simulations and real spaceflight scenarios.

Small sample sizes are another recurring limitation in both HDT and microgravity posture studies, reducing statistical power and generalizability. However, the authors suggest that the use of repeated measures not only enhances statistical power but also allows for the assessment of changes over time, providing more robust and reliable results.

Other analog models also present practical barriers for flavor research. While useful in broader physiological contexts, immersion studies introduce risks such as sample contamination, participant discomfort, and reduced focus during food evaluation, which the authors believe compromise their applicability to flavor research. Similarly, microgravity platforms such as parabolic flights offer only brief exposures to true microgravity (∼20 s), which limits their utility for studying gradual sensory adaptation. These flights are further constrained by the need to select participants who are resistant to motion sickness, which restricts feasibility. However, it is worth noting that parabolic flights offer a valuable analog by combining two critical aspects of spaceflight: short‐term microgravity exposure and a confined, operationally complex environment, both of which contribute to the ecological relevance of such simulations. These environments also provide an opportunity to evaluate the operational feasibility of sample handling, data recording, and participant responsiveness in a realistic space‐like setting. Although short in duration, such flights allow for the testing of immediate sensory responses and food delivery systems that could later be used in orbit.

In contrast, microgravity posture studies offer a cost‐effective and accessible means to investigate postural effects on sensory perception. However, their capacity to simulate fluid shifts akin to those in true microgravity is unclear. Additionally, gravity continues to affect food behavior, potentially influencing swallowing and aroma perception, which should be considered in the study's limitations. While posture‐based models may not replicate all physiological aspects of microgravity, they are valuable platforms to test food handling protocols and posture‐related changes in oral‐somatosensory experience before deploying methods in flight.

Despite these limitations, the authors claim that the physiological analogs remain a promising avenue for flavor perception research. HDT offers a stable platform for observing longer term sensory changes and should be more fully leveraged in future studies. Although parabolic flights present logistical challenges, their ability to simulate true weightlessness offers unique value for real‐time sensory testing. Authors recommend that researchers develop a coordinated strategy that bridges analog and in‐flight testing by validating tools and sample delivery systems stepwise from posture and HDT models to parabolic and orbital platforms. This approach can help standardize protocols for psychophysical evaluation under space constraints. With the emergence of private spaceflight providers (Iles et al. 2023), there is an increasing opportunity to develop controlled experiments that explore short‐term flavor perception changes in actual microgravity environments. Nonetheless, current methods and devices may require modification to be functional and reliable in spaceflight conditions. Their suitability must be evaluated not only for scientific accuracy but also for operational practicality in resource‐limited and weightless environments.

### 4.3 Space Simulations and Relative Merits for Studying Flavor Perception

The eating environment has become increasingly recognized as a critical factor in shaping sensory preferences and experiences. For example, Holthuysen et al. (2017) found no significant differences in liking between a recreated airplane setting and an actual plane, but notable differences emerged when compared to a quiet sensory booth, highlighting the impact of context. Efforts to simulate real‐world contexts have progressed from using sounds, photos, and videos to incorporating Extended Reality (XR) technologies, such as Augmented Reality (AR), VR, Mixed Reality (MR), and immersive screens or experience rooms. These advancements have been extensively reviewed for their ecological validity and potential in sensory research (Crofton et al. 2019; Low et al. 2024; Wang et al. 2021).

Although the application of digital technologies to simulate space conditions remains limited, interest in using these methods for studying flavor perception in space analogs is growing. A total of six published studies on digital simulations related to space content have been identified, utilizing two distinct methods: audio‐ and video‐based simulations (*n* = 2) and VR (*n* = 2). Notably, one study compared the merits of VR and a physiological measure, while another study used a combination of video simulation and microgravity posture. Table 8 summarizes the findings of these studies.

**TABLE 8:** Flavor perception experiments in digital simulations.

Author (year)	Experimental condition	Senses assessed	Key methodology	Key findings
Loke, Chand, et al. 2024	VR (ISS) and neutral context	Odor	*n* = 54; odorants: vanilla, almond, and lemon; odor intensity evaluation on a 5‐point Likert scale (1 = *Not intense at all* to 5 = *Extremely intense*), participant engagement and aroma compound analysis by SPME/GC–MS was conducted.	Significantly higher intensity ratings for vanilla and almond odors in VR, compared to the Neutral context. Those less sensitive to the odors perceived significantly stronger almond odor in VR. GCMS analysis linked these findings to benzaldehyde, a common compound in both vanilla and almond odors.
Low and Loke 2024	Neutral sitting, NASA neutral posture^a^, and VR environment	Odor	Phase 1: *n* = 44; phase 2: *n* = 16 Phase 1: Rated odor intensity on 5‐point Likert scale ratings (odorants: vanilla, lemon, almond) Phase 2: Evaluated emotional responses using SAM and PANAS‐SF scales across contexts and time points in VR (odorants: vanilla, lemon, almond, eucalyptus).^a^, ^b^	No significant differences between postures for odors, but VR caused significant differences for select odors—vanilla and almond. Emotional responses shifted from neutral/positive before VR to more negative after an average of 9 min 35 s in VR.
Loke, Chandrapala, et al. 2024	Microgravity posture and VR^a^	Odor	*n* = 44; intensity of eight odors (vanilla, almond, lemon, lemongrass, lemon myrtle, eucalyptus, peppermint, and vinegar) and control on a 5‐point Likert scale and emotional dimensions.^b^	Higher odor intensity perception in VR for all odors except lemongrass. High odor intensity for almond by high‐sensitivity participants, while high‐intensity perception for vinegar by low‐sensitivity participants in VR.^a^
Gonzalez Viejo, Harris, Tongson, et al. 2024	Normal and simulated microgravity using reclining chairs (positions) with neutral and space‐immersive environments	Taste, aroma, and texture	*n* = 51; samples: six leafy greens—basil, sweet basil, coriander, kale, mixed cos lettuce, and beetroot; affective questions using a 15‐cm nonstructured scale and emotional responses using CATA; biometric responses were collected through videos; univariate and multivariate analyses were conducted to assess self‐reported and biometric consumer sensory analysis.^a^	A significant difference in the room × position interaction for head movements; significant differences in the sample × position interaction for all liking attributes; and nonsignificant differences in the samples × environment interaction.
Tran 2023; Tran and Duizer 2023	ISS environmental sounds at 70 dB versus no sound	Taste	*n* = 60; ISS environmental sounds at 70 dB versus no sound; taste intensity was measured using a 9‐point scale on aqueous solutions in low, medium, and high concentrations (sucrose, salt, citric acid, caffeine, MSG) and chocolate pudding samples.	No statistically significant differences in taste perception across sound conditions for any taste category.
Tran 2023; Tran et al. 2024	Lights on versus lights off during space immersion video with ISS environmental sound		*n* = 29; lights on versus lights off during 20 min space immersion video simulating a rocket launch and Earth orbit; ratings for liking and intensity were collected using a 9‐point hedonic scale and LMS for odor samples (vanilla, almond, citrus), which were delivered at specific intervals (1.5, 5, 10, and 15 min); sensory and emotional responses using CATA and hunger levels were measured before and after the video.	No significant differences in odor intensity across lighting conditions. Liking for citrus increased over time in the “lights off” condition. Emotional responses revealed a decrease in positive emotions and an increase in negative emotions.

Abbreviations: CATA, check that all apply; GC–MS, gas chromatography–mass spectrometry; ISS, International Space Station; LMS, labeled magnitude scale; MSG, monosodium glutamate; PANAS‐SF, Positive and Negative Affect Schedule—Short Form; SAM, Self‐Assessment Manikin scale; SPME, solid‐phase microextraction; VR, Virtual Reality.

^a^ NASA neutral posture mimics “microgravity” posture using a commercial “zero‐gravity” outdoor chair set at 122°–124° (Low and Loke 2024).

^b^ Some participants overlap across different parts of these studies, as they were involved in examining different aspects within the same study.

#### 4.3.1 Audio Simulations (*n* = 1)

Tran (2023) conducted a comprehensive crossover study with 60 participants, investigating the effects of environmental sound (70 dB of actual ISS recordings; NASA's ISS recommended noise level) and no sound on taste perception. The study included two groups (30 participants each) and assessed the five basic tastes—sweet, salty, sour, bitter, and umami—using three concentrations of each taste quality in both aqueous solutions and chocolate puddings as model foods. The results revealed no significant differences in taste perception between the ISS sound and the no‐sound conditions, regardless of taste category or concentration (Tran and Duizer 2023). The findings are interesting as they suggest there may be a more complex interaction between noise level and food perception. For instance, sensory studies investigating environmental white noise levels exceeding 70 dB, including airplane cabin noise, have shown significant effects on taste perception (Lim et al. 2024; Woods et al. 2011; Yan and Dando 2015). This contrast highlights the possibility that noise levels below a certain threshold may have limited sensory impact. Further research is needed to investigate the potential influence of noise intensity and to explore multisensory integration, including the combined effects of sensorial experiences on flavor perception.

#### 4.3.2 Videos and Immersive Screens (*n* = 1)

In a follow‐up study, Tran et al. (2024) explored the influence of contextual factors on odor perception using a 20‐min video simulation of a rocket launch, paired with environmental sounds at 70 dB based on NASA's ISS noise. The study utilizes multiple context‐evoking methods, including written imaginative scenarios and an immersive room setup (screen dimensions: 7.7 × 3.2 m, angled at 126° in the middle to create an illusion of depth). Affective responses (emotion, liking) to three food aromas (vanilla, almond, citrus) were assessed at four time points during the video. Importantly, immersion via screens did not significantly impact aroma perception, suggesting that the combination of visual and auditory stimuli alone may not effectively replicate the sensory demands of spaceship‐related confined environments. The frequency of positive responses dipped slightly and then increased throughout the experiment, while the opposite was observed for the negative emotions. It is possible that responses were task‐related effects (e.g., feeling more positive nearing task completion). This pilot study highlights the importance of considering the duration of testing for emotional responses to emerge. However, the lack of significant effects raises questions about the utility of screens and sound alone for sensory simulations in space‐related tasks.

#### 4.3.3 VR Simulations (*n* = 2)

Loke, Chand, et al. (2024) conducted a study with 54 participants to examine food odor perception in a virtual spacecraft environment. Participants evaluated the intensity of three odors (vanilla, almond, and lemon) in two contexts: a neutral context and a VR simulation replicating the ISS. The VR environment featured floating objects, operational sounds to mimic space conditions, and an integrated questionnaire in the VR itself. Odor intensity was rated on a 5‐point Likert scale for one concentration, revealing significantly higher intensity for vanilla and almond in VR, while lemon odor remained consistent across contexts. Further analysis revealed that participants with lower baseline odor sensitivity perceived stronger almond and vanilla odors in VR in general. Interestingly, the authors suggest that the increased perception of almond and vanilla odors was likely due to the presence of benzaldehyde, a volatile compound detected in both these odors during GC–MS analysis. While benzaldehyde is not typically found in commercial vanilla extracts, its presence was likely due to its use as a food additive.

In a follow‐up pilot study exploring the duration required for individuals to perceive feelings of isolation within a VR environment, Low and Loke (2024) investigated odor intensity and emotional responses among 16 participants. Participants were exposed to four distinct odors (vanilla, almond, lemon, and eucalyptus) and evaluated these at two time points. Participants remained in the VR environment as long as possible until they began to feel isolated, at which point the second evaluation occurred. The findings revealed that increased odor intensity perception and emotional responses, initially neutral or positive (e.g., “interested,” “attentive”), shifted to more negative emotion terms (e.g., “nervous,” “guilty”) after spending approximately 9 min and 35 s in the VR environment. Importantly, the study also identified clusters of participants based on their emotional regulation and reactions to the VR experience, highlighting significant individual variation in how participants perceive and respond to isolation in a VR spaceship context. This demonstrates VR's potential as a ground‐based analog for sensory research but a need to account for individual variation in how they interact with VR tools, by evoking both sensory and emotional experiences related to food and highlighting its utility in advancing sensory research.

#### 4.3.4 Combination/Comparison of Digital Tools and Postures (*n* = 2)

Acknowledging the importance of combining simulation tools, Loke, Chandrapala, et al. (2024) compared odor perception between microgravity posture and VR simulation, to understand the variation in odor intensity for eight odors including vanilla, almond, peppermint, vinegar, eucalyptus, lemon, lemon myrtle, and lemongrass. Their study demonstrated that odor intensity was significantly heightened in the VR environment compared to the microgravity posture for most odors. Notably, polarizing, trigeminal (e.g., eucalyptus, peppermint, vinegar), and citrus (e.g., lemon, lemon myrtle) aromas were perceived more intensely in VR, likely due to shared chemical attributes identified through GC–MS analysis (e.g., benzaldehyde in almond and vanilla extracts) and trigeminal properties (e.g., menthol in peppermint). In contrast, floral odors like lemongrass showed no difference across contexts, possibly due to their simpler volatile profiles (Loke, Chandrapala, et al. 2024). The authors emphasized that both approaches were different; while the microgravity posture replicates the physical aspects of space conditions, VR provides immersive visual–spatial cues. However, neither method alone fully replicates the comprehensive immersive experience of actual microgravity. The authors proposed an integrated approach for a more engaging space‐like simulation, but technological constraints of VR at the time prevented the methods from being combined.

More recently, Gonzalez Viejo, Harris, Tongson, et al. (2024) combined microgravity posture with 180° video simulations to explore sensory perception in space‐like environments. Fifty‐one participants evaluated six leafy greens (e.g., kale, lettuce, basil) across four contexts, assessing aroma, texture, and overall liking, alongside biometric and facial expression measures. While the study found minimal differences across contexts, head movements varied when screens were used with the microgravity posture, possibly due to unconscious tilting in response to the immersive setup lacking ceiling projections, despite instructions to focus on the screen. For astronauts, sensory perception is shaped by physiological changes and the isolation of space, as discussed in Section 4.2. Although the combination of microgravity postures and space‐themed video simulations elicited mixed emotional responses due to the novelty of the setup, this highlights a key limitation. Earth‐based simulations with microgravity posture chairs and projection screens alone may not replicate the multidimensional sensory and psychological challenges of real space‐living environments, where the confined and cluttered conditions of a space shuttle introduce additional stressors that may explain discrepancies in findings.

An interesting study not focused on food‐related sensory perception but relevant to posture in immersive environments is that of Jiang, Gong, et al. (2023) and Jiang, Fang, et al. (2023). They examined the effects of HDT position (body fluid movements) rather than microgravity posture chairs. Their findings demonstrated that VR combined with varied postures, such as −12° HDT and 9.6° head‐up tilt (seated for up to 3 h prior to evaluation), significantly influenced cognitive performance, emotional states, perceptions of environmental color settings, and visual tracking. Notably, HDT reduced task performance and positive emotional responses across different environmental color conditions (Jiang, Gong, et al. 2023) and reduced visual tracking accuracy (Jiang, Fang, et al. 2023), highlighting the importance of accounting for postural effects in immersive sensory studies. However, the integration of HDT with other environmental or social factors, particularly in the context of flavor perception, remains unexplored.

#### 4.3.5 Immersive Screens or VR to Simulate Space‐Like Eating Environment?

Both immersive screens and VR simulations offer valuable opportunities for replicating space‐like environments in sensory research, particularly for food‐ and sensory‐related studies. However, these tools differ significantly in their ability to evoke sensory and emotional responses, necessitating careful consideration of the research objectives when selecting between them. VR appears to offer stronger emotional and sensory engagement for environments aimed at simulating confined or stressful space conditions. Studies demonstrate that VR can evoke evolving emotional states over time, such as *nervousness* and *loneliness*, which may align closely with psychological challenges associated with space‐like confinement (Low and Loke 2024). In contrast, immersive screens tend to produce more stable emotional responses, typically neutral or positive responses (Gonzalez Viejo, Harris, Tongson, et al. 2024; Tran 2023). While this distinction suggests different applications, further research is required to determine the long‐term effects of both methods in space simulations.

VR's heightened engagement also extends to sensory responses. For instance, odors such as almond and vinegar elicited varying emotional and sensory outcomes during VR exposure, with positive emotions amplifying odor perception and stress dampening the perception of polarizing scents like vinegar (Loke, Chandrapala, et al. 2024). Notably, prolonged VR exposure led to shifts toward negative emotional states, highlighting the importance of temporal dynamics when simulating sensory and emotional experiences. A significant limitation of both methods, however, is the potential influence of novelty or unfamiliarity with simulated environments on participants’ responses. This raises questions about whether the observed emotional and sensory outcomes truly reflect space‐like contexts or are instead driven by initial exposure effects. Given that astronauts undergo extensive training and may experience adaptation over time, future research should explore how prolonged exposure to these simulations influences sensory perception and psychological states. Additionally, handling food products in VR versus immersive screen environments presents a practical consideration. The way individuals interact with food, utensils, and packaging may differ significantly between the two methods, potentially influencing sensory experiences. Future research should address this by incorporating repeated VR exposures to explore the long‐term effects of familiarity. Additionally, validating these findings through comparisons with established analogs, such as long‐term terrestrial confinement studies, would enhance their ecological validity and strengthen their applicability for space research.

#### 4.3.6 Key Insights and Recommendations: Flavor Perception Research Using Digital Simulations

In the authors’ view, the focus of many studies has been limited to odor perception due to its simplicity, neglecting the complex integration of taste and smell required for real food‐based sensory evaluations. Additionally, there is a limited understanding of how varying concentrations of flavor compounds impact sensory perception in digital simulations. This limitation prevents a comprehensive understanding of how digital simulations can replicate real space flavor experiences.

Moreover, current studies often employ VR sessions lasting under 10 min, which may be insufficient to account for individual variability in sensory responses. The lack of standardized protocols for VR exposure times limits the reliability of sensory findings and their applicability to confined environments. In addition, many VR simulations use low‐resolution, low‐polygon models of environments of ISS, which reduces the immersive quality of the experience, potentially diminishing the accuracy of sensory evaluations. The authors suggest that upgrading VR simulations to enhance the actual spaceship environment could enhance immersion and isolation. This may include integrating real‐time environmental variables (e.g., soundscape, light control, tactile feedback) to more accurately mimic the sensory demands of long‐duration spaceflight.

Most studies lack direct comparisons to actual space environments, relying instead on hypotheses drawn from prior sensory‐context research. While these studies highlight the ecological validity of using context to evaluate sensory responses, the authors argue that it remains unclear whether the sensory reactions observed in simulated environments accurately replicate those experienced in real confined or space‐like conditions. An additional challenge in VR‐based flavor perception research is the limited ability to interact with and visualize food in a realistic manner, which may influence sensory expectations and perceived flavor attributes. The authors recommend that developing improved haptic feedback and interaction systems could enhance the realism of food‐related tasks within digital simulations.

To address these limitations and advance the field, the authors suggest that incorporating actual foods and beverages into VR simulations is essential to explore the interplay between taste and smell comprehensively. Research should also explore food consumption experiences (i.e., more realistic eating experience) within VR to better simulate real‐world sensory interactions in confined environments. As part of this, testing different food delivery methods such as straw‐fed pouches or pre‐portioned trays within VR contexts may help identify mechanisms that can be adapted across simulation platforms and, eventually, in actual spaceflight. Furthermore, while posture has been shown to influence sensory responses in video‐based simulations, its role in VR remains underexplored. Studies should build on evidence from video‐based simulations to explore how posture interacts with VR in flavor perception. In the authors’ view, combining immersive environments with varied body positions, such as head‐down tilt postures (body fluid movements), could provide additional insights into sensory experiences under space‐like conditions.

From the authors’ perspective, when evaluating food‐related emotional responses in digital simulations of space environments, greater emphasis should be placed on cognitive and behavioral responses rather than relying solely on physiological indicators. While biometric tools can provide supplementary insights, current evidence suggests they may not reliably capture emotional or hedonic responses in immersive sensory studies independently of explicit measures (see systematic review, Low et al. 2022), particularly in contexts involving complex stimuli such as food. This limitation becomes even more pronounced in extreme or altered environments, such as space or its analogs, where physiological changes unrelated to flavor perception may confound biometric data. Therefore, the reliability and interpretive validity of biometric measures in these settings warrant careful re‐evaluation. The integration of implicit and explicit measures can be valuable, but only when supported by well‐controlled, contextually sensitive study designs (an area requiring additional validation). Moreover, although VR‐based tools hold significant promise for simulating confined environments, their current hardware and interface capabilities may require adaptation for use in low‐gravity conditions. Effectiveness should be validated not only in terms of perceptual realism but also in terms of operational usability within microgravity or analog scenarios. Finally, the selection of methods, tools, and experimental designs should be determined by the specific research aims, resource constraints, and the required degree of environmental fidelity.

## 5 Recommendations and Concluding Remarks

This review examines flavor perception in spaceflights, space analogs, and digital simulations, highlighting key advancements commensurate with an increased number of people traveling into space and an increase in spaceflight mission duration. Ongoing challenges have also been identified. While many studies have explored individual factors such as microgravity or confinement, the interplay between physiological, psychological, and environmental influences on sensory experiences remains insufficiently understood. Addressing these gaps is crucial for developing effective sensory strategies for long‐duration missions.

Flavor perception in space is closely linked to astronaut health and well‐being, as sensory changes can affect food enjoyment, appetite, and overall nutrition. Understanding these changes can guide the development of space‐friendly foods, menus, and feeding systems, ensuring optimal dietary intake and psychological well‐being. Additionally, preflight sensory optimization can help design meals that retain their appeal under space conditions. Building on these insights, future research could focus on the following key areas:

- Enhancing simulation realism: Integrating VR with physiological simulations or parabolic flights offers a potential method for replicating space‐like conditions. VR can recreate spatial and visual elements, while parabolic flights provide brief microgravity exposure. However, the feasibility of VR‐based sensory assessments in parabolic flights is uncertain due to physical constraints and the short duration of weightlessness, which may limit ecological validity, particularly given the role of adaptation and familiarity in sensory perception.
- Improving ecological validity in sensory studies: Future research should focus on ergonomic, reclined‐compatible VR systems to enhance space simulation realism. Mixed‐reality tools with passthrough cameras could enable natural food interactions, while incorporating social elements, such as viewing crew members in VR, may provide insights into the role of communal dining in sensory perception and food enjoyment. Additionally, studies should compare responses between unfamiliar confined and cluttered VR environments and familiar VR settings, as most participants have never experienced space. Investigating cognitive and perceptual differences between these conditions could help distinguish true space‐like context responses from novelty effects.
- Targeted testing with relevant populations: Sensory studies conducted in Earth‐based analogs often rely on general populations, which may not accurately reflect the sensory responses of astronauts. Given their extensive training, physiological adaptations, and unique psychological profiles, astronauts (or astronaut candidates) may react differently to sensory stimuli in space‐like conditions. Future studies should focus on recruiting trained individuals to ensure findings apply to space missions, at least until affordable space tourism expands research opportunities.
- Establishing standardized sensory testing methodologies: A lack of consistent protocols for sensory testing has limited the comparability of findings across studies. Future research should clearly define the type of sensory methods, appropriate scales, and sample formats. They should also be suited to posture and microgravity constraints. Data collection should use compact digital tools, and designs should include repeated measures to track changes over time. Establishing standardized sensory testing methodologies applicable to both spaceflights and analogs is essential to generate reliable data and support future advancements.

These recommendations underscore the importance of interdisciplinary approaches that combine advanced technologies, innovative methodologies, and standardized protocols to enhance the ecological validity of research in this domain. While advancements in space analogs and simulations have provided valuable insights, there is a need to address the limitations of current methodologies. A more comprehensive and integrative approach will be crucial to understanding flavor perception in space environments and to optimizing food systems that support astronauts’ sensory and nutritional needs during missions.

## Author Contributions

**Thejani Prabodha**: conceptualization, methodology, investigation, validation, visualization, writing – original draft, formal analysis, data curation. **Lukas Danner**: conceptualization, investigation, writing – review and editing, methodology, supervision. **Gail N. Iles**: conceptualization, funding acquisition, investigation, writing – review and editing, supervision. **Martina Heer**: writing – review and editing, investigation, validation. **Isabelle Mack**: writing – review and editing, investigation, validation. **Charles Brennan**: funding acquisition, supervision, writing – review and editing. **Julia Low**: conceptualization, investigation, funding acquisition, writing – original draft, methodology, validation, writing – review and editing, project administration, supervision.

## Conflicts of Interest

The authors declare no conflicts of interest.

## Supporting information




**Supplementary Materials**: crf370241‐sup‐0001‐SuppMat.docx
