# Supplementary material for: Initiating Sensory Science Research in Space Conditions—Current Practices and Future Perspectives
Source: Compr Rev Food Sci Food Saf. 2025 Aug 3;24(5):e70241. doi: 10.1111/1541-4337.70241 (PMC12319303; doi:10.1111/1541-4337.70241)
Supplement: Supplementary file 1 — Supplementary Materials: crf370241‐sup‐0001‐SuppMat.docx [file CRF3-24-e70241-s001.docx]

**Supplementary Materials A**

***Overview of space travel conditions on physiological responses***

Space radiation, isolation and confinement, distance from Earth, gravity changes, and hostile/closed environments (Childress et al., 2023)are the major hazards that collectively pose challenges to human space exploration while affecting sensory aspects of food perception (Douglas et al., 2016; Douglas et al., 2021; Milon et al., 1996; Šolcová et al., 2016; Stuster, 2016; Vessel & Russo, 2015; Watkins et al., 2022; Zwart et al., 2009). Given the paramount importance of addressing challenges related to altered gravity and isolation and confinement on sensory perception, especially their interaction with the complex biological and psychological factors affecting sensory perception, our focus is on understanding how eating related sensory perception varies under these conditions.

Microgravity refers to the condition of near weightlessness (Beysens & Van Loon, 2015; Goswami et al., 2021) experienced during spaceflight due to reduced gravitational forces. This environment induces physiological changes such as fluid redistribution and physiological adaptation in astronauts. On Earth, the hydrostatic pressure of the blood column leads to fluid accumulation in the legs due to gravity, creating a hydrostatic gradient along the body axis. The specific part of the human vascular system that experiences constant hydrostatic pressure in terrestrial gravity is referred to as the hydrostatically indifferent point (HIP) of the body (Noskov, 2013). In microgravity or simulated microgravity conditions, the absence of this gradient causes blood redistribution from the legs towards the chest and head, leading to an increase in heart volume and cardiac output. This redistribution can result in symptoms such as redout, edema, and hyperemia in the upper body. Edema in facial and neck tissues may occur, affecting astronauts' well-being and efficiency (Noskov, 2013). As a consequence of these mechanisms, astronauts may experience edema in the facial and neck tissues, leading to a decline in their overall well-being and efficiency. Despite these challenges, the human body adapts to microgravity over time (Antonutto & Di Prampero, 2003; Baker et al., 2019; Iwase et al., 2020; Norsk, 2020), although its effects on different organ systems vary for example musculoskeletal (Comfort et al., 2021; Lackner & Graybiel, 1979), vestibular (Carriot et al., 2021), cardiovascular (Baran et al., 2021), nervous (Cassady et al., 2016; Clement et al., 2020; Hupfeld et al., 2021), neuro-ophthalmology (Lee et al., 2020; Ong et al., 2023), endocrine (Leach et al., 1988), and digestive systems (Yang et al., 2020), influencing overall health and performance during and after space missions. The accumulation of excess fluid in the nasal passages during space flight can lead to a decrease in airflow caused by the engorgement of the non-olfactory mucosa resulting reduced perception of flavour in foods (Olabi et al., 2002) reducing the food intake, which may potentially impact on the insufficient nutrient intake. Moreover, during these missions, crew members face physiological (Pagel & Choukèr, 2016; Schmitt & Schaffar, 1993) and psychological challenges (Kanas & Manzey, 2008; Palinkas, 1992; Palinkas et al., 2004) due to isolation and confinement, which can lead to cognitive and behavioural issues (Gemignani et al., 2014; Palinkas et al., 2004), mood swings (Basner et al., 2014; Gemignani et al., 2014; Kanas, 1990; Schneider et al., 2010), and nutritional (Zwart et al., 2021) and metabolic changes (Pagel & Choukèr, 2016; Strollo et al., 2018). The significance of food familiarity, choice, and communal dining experiences becomes more pronounced, as food serves as a crucial source of comfort and social interaction in isolated
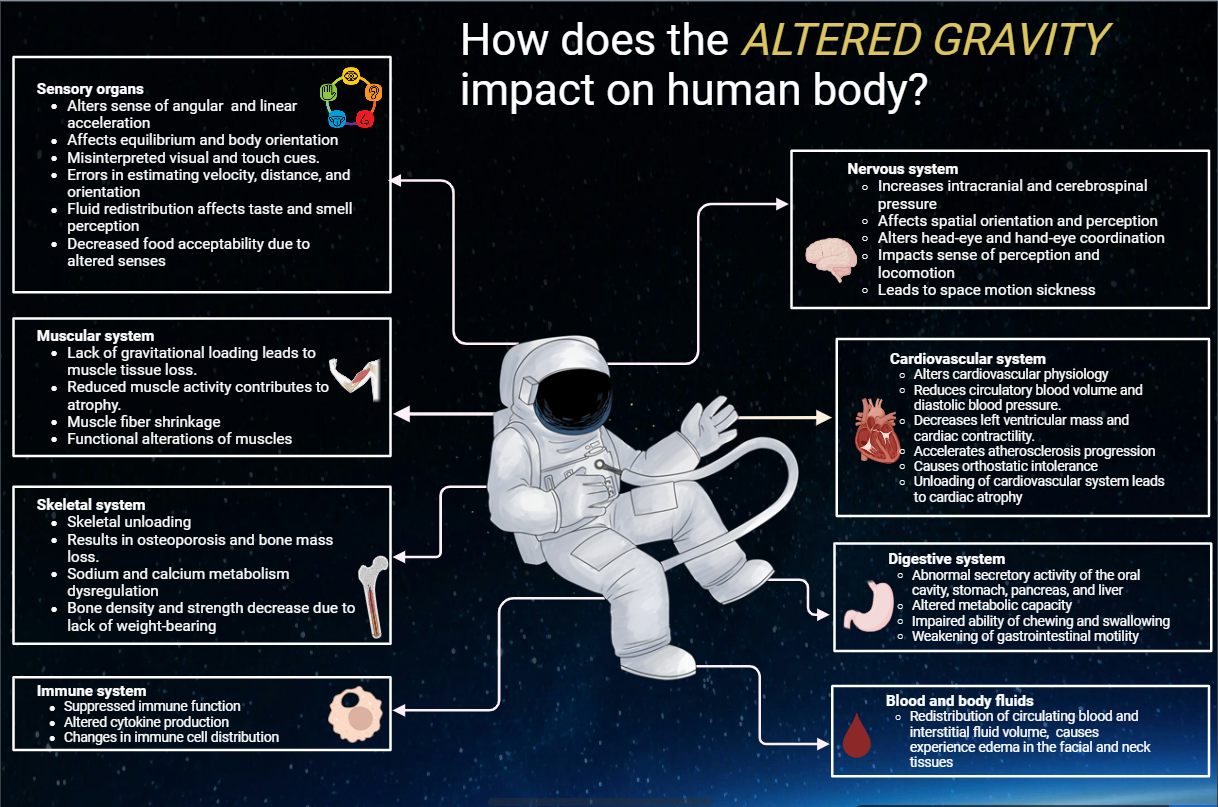
environments (Stuster, 2007).

**Figure 1.** Physiological responses to changes in gravity summarising the effects of altered gravity on the human body, including sensory disruptions, muscle atrophy, skeletal deterioration, cardiovascular changes, digestive impairments, immune suppression, and fluid redistribution. The impact on each system is further detailed.

**Supplementary Materials B**

***Table 1. Brief Overview and Description of Space Analogue and Space Simulation Studies Including Applicability to Study Flavour Perception^1^***

| **Space Analogue and Simulation Methods (Abbreviation, if available)** | **Picture** | **Description** | **Applicability to Study Flavour Perception^2,3^** |
| --- | --- | --- | --- |
| Clinostat (1D/ 2D) | 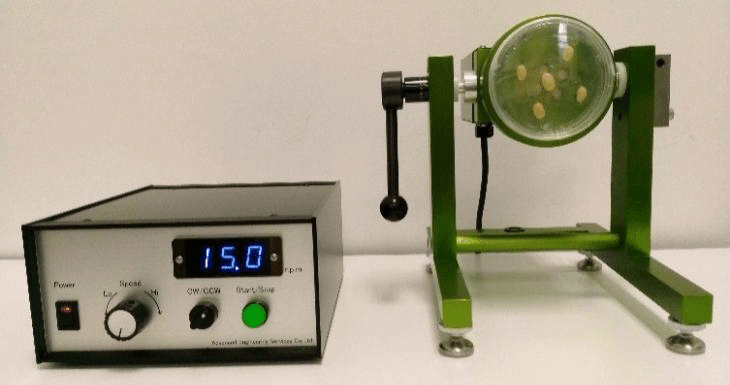Figure 2. Clinostat [source: Palma Jiménez et al. (2017)] | Method: Reduced gravity (≤10^-3^g)  Application: Cells, microbes, plants  Experiment duration: Hours to weeks | No |
| Clinostat (3D) | 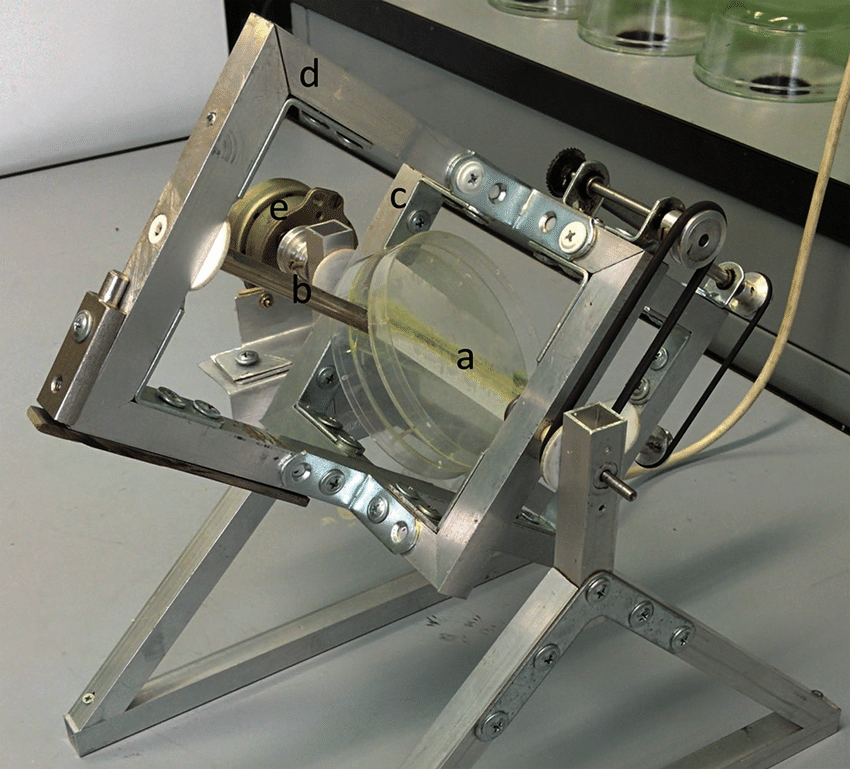Figure 3. Clinostat [source: Veronika et al. (2019)] | Method: Reduced gravity (10^-4^g)  Application: Cells, microbes, plants  Experiment duration: Hours to weeks | No |
| Rotating Wall Vessel (RWV) | 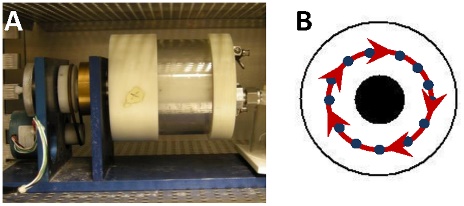Figure 4. The Rotating Wall Vessel [source: Edsall and Franz-Odendaal (2014)] | Method: Reduced gravity (≤10^-3^g)  Application: Cells, microbes, plants  Experiment duration: Hours to weeks | No |
| Random Positioning Machine (RPM) | 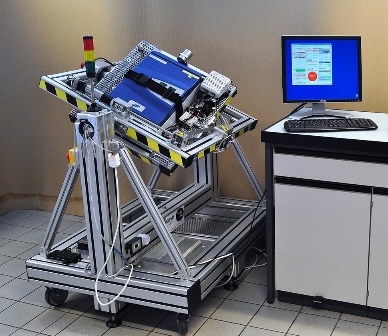Figure 5. Random Positioning Machine [Source: Franco-Obregón et al. (2018)] | Method: Reduced gravity (10^-4^g)  Application: Cells, microbes, plants  Experiment duration: Hours to weeks | No |
| Diamagnetic Levitation | 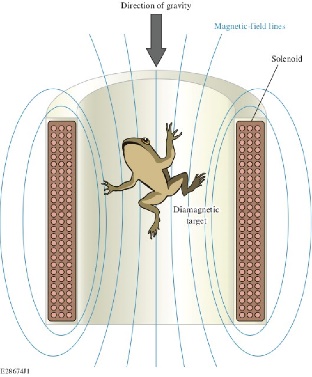Figure 6. Diamagnetic levitation configuration [Source: Bruhaug and Beveridge (2020)] | Method: Reduced gravity (<10^−2^ g)  Application: Cells, microbes,  plants, animals  Experiment duration: Minutes to hours | No |
| Bed rest | 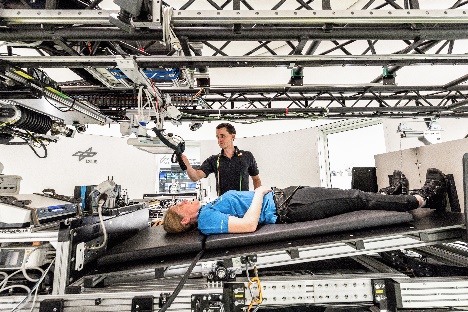Figure 7. Head down Tilt bed rest [Source: NASA (2023b)] | Method: Hypo-gravity  Application: Humans  Experiment duration: Hours to months | Yes |
| Unilateral lower limb suspension (ULLS) | 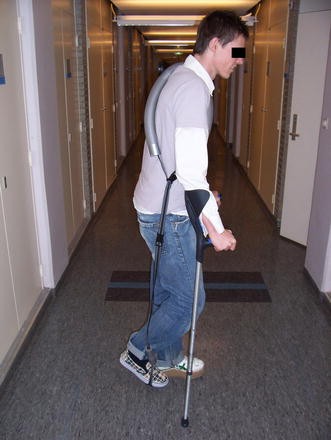Figure 8. Unilateral lower limb suspension [Source: Horstman et al. (2012)] | Method: Microgravity  Application: Humans  Experiment duration: Hours to weeks | No |
| The Rodent Hindlimb Unloading (HU) | 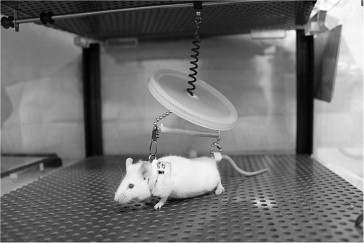Figure 9. The Rodent Hindlimb Unloading [Source: Wilson et al. (2012)] | Method: Microgravity  Application: Animals  Experiment duration: Hours to weeks | No |
| Neutral buoyancy | 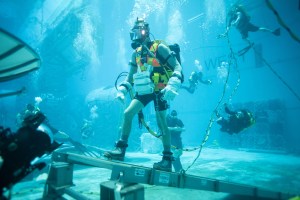Figure 10. Neutral buoyancy [Source: NASA (2024b)] | Method: Microgravity  Application: Animals, humans  Experiment duration: Hours | No |
| Wet immersion | 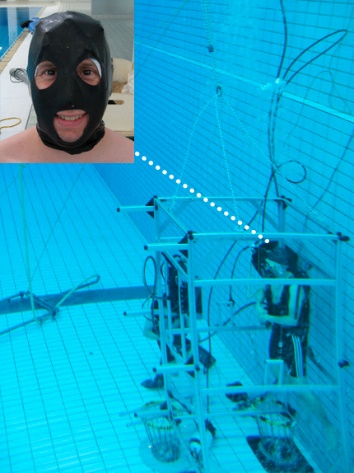Figure 11. Wet immersion [Source: Schneider et al. (2014)] | Method: Microgravity  Application: Humans  Experiment duration: 6-12 hrs | No |
| Dry immersion | 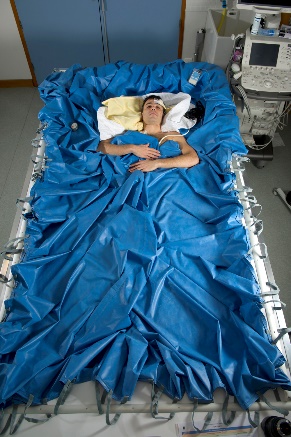  Figure 12. Dry immersion [Source: MEDES (2023)] | Method: Microgravity  Application: Humans  Experiment duration: Days to month | Yes |
| Parabolic flight | Figure 13. Parabolic flight [source: NASA (2023b)] 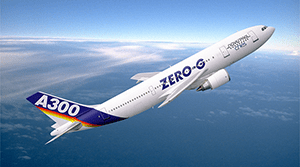 | Method: Microgravity (10^− 3^ - 10^− 2^g)  Application: Cells, microbes, plants, animals,  humans  Experiment duration: Seconds (15-20s) | Yes |
| Drop towers | Figure 13. 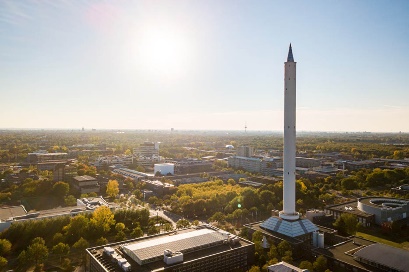Drop towers [Source: ESA (n.d-b)] | Method: Microgravity (10^− 5^ – 10^− 2^g)  Application: Cells, microbes, plants  Experiment duration: Seconds (2-9s) | No |
| Sounding rockets | 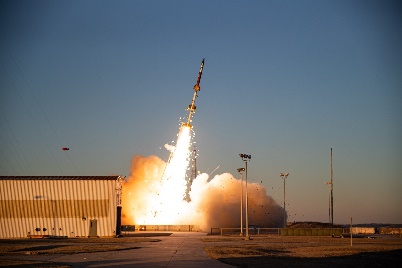Figure 14. Sounding rockets [Source: NASA (2023a)] | Method: Microgravity (10^− 4^ - 10^− 3^g)  Application: Cells, plants, microbes, animals  Experiment duration: Minutes (5-20 mins) | No |
| Microgravity posture chair | 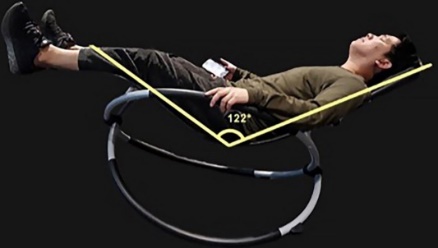Figure 15. Microgravity posture [source: Loke et al. (2024b)] | Method: Microgravity posture  Application: Humans  Experiment duration: minutes | Yes |
| Isolated, Confined and Extreme (ICE) environments | 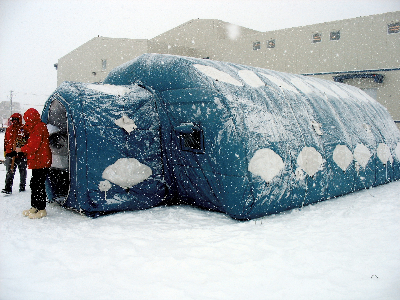Figure 16. Antarctic station [source: NASA (2023b)] | Method: Isolation and confinement  Application: Humans  Experiment duration: Months-years | Yes |
| Isolated, Confined and Controlled (ICC) environments | 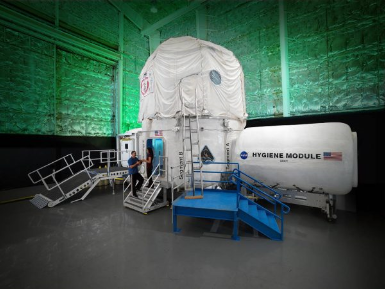Figure 17. HERA [source: NASA (2023b)] | Method: Isolation and confinement  Application: Humans  Experiment duration: Months-years | Yes |
| Space video Simulations | Figure 18. Simulated ‘space’ u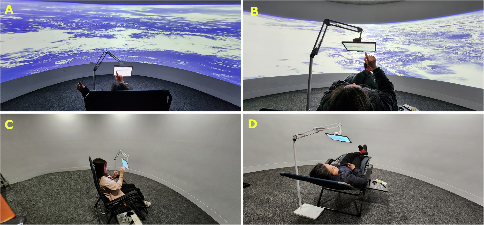sing video simulation [source: Gonzalez Viejo et al. (2024b)] | Method: Digital simulation of space environment  Application: Humans  Experiment duration: minutes - hours | Yes |
| Virtual Reality simulations of space | 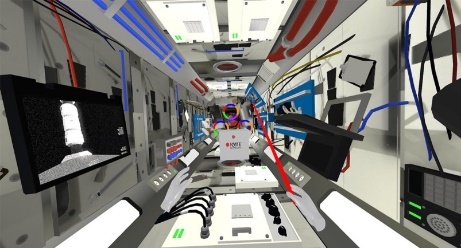Figure 19. Simulated ‘space laboratory’ utilised during the VR [source: Loke et al. (2024b)] | Method: Digital simulation of space environment  Application: Humans  Experiment duration: minutes | Yes |
| ^1^ Table adapted from Ferranti et al. (2020) and Oluwafemi and Neduncheran (2022)  ^2^ Applicability to study flavour perception based on realistic requirements of human sensory-related experiments, such as the ability to obtain quantitative data on sensory effects and participant interaction with stimuli (e.g., tastants, odorants, actual food samples, or viewing food-related videos/pictures). Yes/No  ^3^ If "Yes" to the above, indicate the senses applicable for study: taste, smell, visual (texture/colour), sound (texture), touch, and trigeminal senses. | | | |

**Supplementary Materials C**

***Table 2. Isolation and confinement analogues for Human research partnered by Space agencies^1,2^***

| Analogue mission | Location | Type of environment | Mission Duration | Crew size | Specific characteristics | Study areas | Reference |
| --- | --- | --- | --- | --- | --- | --- | --- |
| Isolated Confined and Extreme (ICE) environments | | | | | | | |
| Arctic missions | Haughton-Mars Project Research Station (HMP RS)  Devon Island, Arctic Canada. | Hazzard: Isolation and confinement  Environment: desert | Every summer since 1997 | 4-13 | Field with barren terrain, freezing temperatures, and isolation which is located on the world’s largest uninhabited island and mimics the environmental conditions on Mars and other planets. | To develop, test, and validate new exploration technologies and strategies for planning the future human and robotic exploration of the Moon and Mars. | NASA (2023b), de Weck and Simchi-Levi (2006); Lee (2002); Seitz and Glass (2017) |
| Antarctic Stations | McMurdo Station (U.S. station) | Hazzard: Isolation and confinement  Environment: Closed habitat | 3-6 months/ Winter-over | 250-1000 | Extreme environment with day/night variations which provides a unique and accessible test bed to develop prototype systems and technologies for use on the Moon and Mars. | Astronomy, atmospheric sciences, biology, Earth science, environmental science, geology, glaciology, marine biology, oceanography, climate studies, and geophysics. | NASA (2023b); NSF (2024) |
|  | Palmer Station (U.S. station) | Hazzard: Isolation and confinement  Environment: Closed habitat | 3-6 months/ Winter-over | 20-44 | A site that is accessed routinely during the winter consists of well-equipped laboratory | For study of Biological, Ornithological, Meteorological, Atmospheric, Glaciological and Marine Ecosystem science. | NASA (2023b); NSF (2024) |
|  | Concordia mission, Antarctica | Hazzard: Isolation and confinement  Environment: Closed habitat | < 4 months (summer and 12 months winter over projects) since 2005 | 16 | Consists of two towers one for machinery and the other for living quarters. The site has irregular sunlight, no immediate rescue, extremely low temperatures and little oxygen in the air. | Glaciology, atmospheric studies, astronomy, human-related research, psychology, physiology, and medicine and technologies. | NASA (2023b) |
|  | Neumayer-Station III | Hazzard: Isolation and confinement  Environment: Closed habitat | Year-round since 1981 | 40-60 | Integrates research, operational and accommodation facilities. Extreme environment with day/night variations | Meteorology, air chemistry and geophysics, biology and environmental studies | AWI (2022) |
| NASA Extreme Environment Mission Operations Project (NEEMO) | 5.6 kilometres (3.5 miles) off Key Largo in the Florida Keys National Marine Sanctuary | Hazzard: isolation and confinement, remote locations  Environment: hostile, extreme environment | < 3 weeks |  | The use of a technique known as saturation diving allows the aquanauts to live and work underwater for days or weeks at a time | To simulate living on a spacecraft and test spacewalk techniques for future space missions. Studying behavioural stressors, team cohesion, crew autonomy, circadian rhythms, nutritional supplementation, and immune function and technology demonstrations | (NASA, 2023b) |
| Isolated Confined and Controlled (ICC) environments | | | | | | | |
| Human Exploration Research Analogue (HERA) | Johnson Space Center, Houston, Texas | Hazzard: Isolation, confinement  Environment: Closed and remote environment | Up to 45 Days since 1964 (Russian academy of science) | 4 | 650-square-foot habitat split among two floors and a loft lofted habitat module with airlock and hygiene module | Behavioural health and performance assessments, communication and autonomy studies, human factors evaluations, and medical capabilities assessments | NASA (2023b); NASA (2024a) |
| Nezemnyy Eksperimental’nyy Kompleks (NEK) | IBMP Moscow. | Hazzard: isolation, confinement, and remote conditions | More than  1 year since 1960 | 6 | A pressurised multi-compartment facility that can accommodate large international crews and operate for a long duration | Immunology, physiology, microbiology, metabolic studies, telemedicine, cognitive and behavioural health, and team dynamics  Notable studies: SIRIUS (120 days)  Mars 500 Three missions 14-day, 105-day and 520-day (2007-2011) | NASA (2024c) |
| JAXA Isolation Chamber | JAXA's Tsukuba Space Center | Hazzard: Isolation, confinement  Environment: Closed and isolated environment | < 6 months |  | Consists of two connected cylindrical rooms, each measuring 11 meters long by 3.8 meters wide by 2 meters tall, beds for eight people | Effects of mental and phycological stresses | JAXA (2008) |
| Human Exploration System Testbed for Integration and Advancement (HESTIA) | Johnson Space Center | Environment: Closed-Loop Atmospheric Representation | < 90 days since 2015 |  | The only NASA facility that has the unique experience, chamber geometry, infrastructure, and support systems capable of conducting deep-space Environmental Control & Life Support System research. Can operate at reduced pressure and elevated oxygen environments. | Environmental Control & Life Support System (ECLSS), Habitation Systems, Human Health and Performance, Human Research Program (HRP) | Marmolejo and Ewert (2016); NASA (2023b) |
| Crew Health and Performance Exploration Analogue (CHAPEA) | Johnson Space Center | Hazzard: resource limitations, isolation, equipment failure, and significant workloads | < one year |  | simulates a realistic Mars habitat to support long-duration, exploration-class space missions with 3D printed habitats including private crew quarters, a kitchen, and dedicated areas for medical, recreation, fitness, work, and crop growth activities. | To characterise the risk of the planned exploration food system design about crew health and performance and inform NASA standards, associated vehicle mass and volume requirements, and resource-risk trades for long-duration exploration missions. | NASA (2023b) |
| Human-Rated Altitude Chamber Complex (ACC) | Johnson Space Centre | Environnent : confinement and hostile environnement | < 91 days |  | Consists of eight experimental altitude chambers which are configured for a particular type of testing consisting of manned and unmanned test environments. | Development, certification, and parametric testing of life  support systems for humans in the hostile environment of space | NASA (2023b) |

^1^ Space agencies include the organisations responsible for space exploration, research, and technology development. NASA (National Aeronautics and Space Administration), Roscosmos (Russian Federal Space Agency), ESA (European Space Agency), JAXA (Japan Aerospace Exploration Agency), and CSA (Canadian Space Agency), which contribute to understanding space and advancing technologies to benefit Earth.

^2^ These methods have the potential to be applied to food-related research, although they have not yet been explored in this context. The applicability of food-related research refers to the examination of how different factors influence sensory experiences such as taste, smell, and flavour perception. This includes psychophysical, psychological, and social aspects. The psychophysical aspect covers changes in sensory intensity, where external conditions can lead to increases or decreases in the perception of flavours. The psychological aspect addresses how mental states like stress, isolation, or mood impact sensory perception, potentially altering the way individuals experience taste or smell. The social aspect examines how social interactions, or the lack thereof, influence sensory experiences, affecting food preferences.

***Table 3. Microgravity analogues for Human research partnered by Space agencies^1,2^***

| Analogue mission | Location | Type of environment | Mission Duration | Crew size | Specific characteristics | Study areas | Reference |
| --- | --- | --- | --- | --- | --- | --- | --- |
|  |  |  |  |  |  |  |  |
| DLR  :envihab | Cologne, Germany | Bed Rest Study | < 100 days |  | Eight modules include a short-arm human centrifuge, laboratories for studying the effects of oxygen reduction and pressure decrease, rooms for psychological stress simulations and rehabilitations, and microbiological and molecular biological research tools. | Studies of musculoskeletal, cardiovascular deconditioning and psychological effects of long-term confinement to a reduced gravity environment. | DLR (n.d); NASA (2023b) |
| MEDES (Institute for Space Medicine and Physiology) | Toulouse, France | Bed Rest Study and dry immersion | Bed rest- Weeks to months  Dry immersion:7-14 days | 2-24 | Consists of 14 beds, a short-arm centrifuge used to evaluate the use of artificial gravity as a countermeasure for weightlessness-induced disorders.  Equipped with two dry immersion bathtubs | Studies of musculoskeletal, cardiovascular deconditioning and psychological effects of long-term confinement to a reduced gravity environment. | MEDES (2023) |
| IBMP | IBMP  Moscow, Russian | Dry immersion | 7-14 days |  | Two immersion tanks with medical support and imaging | Studying physiological changes in musculoskeletal, cardiovascular, and sensorimotor systems | ESA (n.d-a) |
| Parabolic flight | The Johnson Space Center | Microgravity | 22 seconds |  | Involves an aircraft repeatedly flying in a pattern where it changes altitude and speed. The vertical load factor goes from 1.8g to zero gravity for about 22 seconds. A single flight typically consists of 30-60 parabolas | for studying human physiological function, operational training, and procedures that are relevant to orbital and deep-space missions | NASA (2023b) |

^1^ Space agencies include the organisations responsible for space exploration, research, and technology development. NASA (National Aeronautics and Space Administration), Roscosmos (Russian Federal Space Agency), ESA (European Space Agency), JAXA (Japan Aerospace Exploration Agency), and CSA (Canadian Space Agency), which contribute to understanding space and advancing technologies to benefit Earth.

^2^ These methods have the potential to be applied to food-related research, although they have not yet been explored in this context. The applicability of food-related research refers to the examination of how different factors influence sensory experiences such as taste, smell, and flavour perception. This includes psychophysical, psychological, and social aspects. The psychophysical aspect covers changes in sensory intensity, where external conditions can lead to increases or decreases in the perception of flavours. The psychological aspect addresses how mental states like stress, isolation, or mood impact sensory perception, potentially altering the way individuals experience taste or smell. The social aspect examines how social interactions, or the lack thereof, influence sensory experiences, affecting food preferences.

**Supplementary materials D**

***Table 4. Key standards and guidelines for sensory evaluation for consideration in space-related studies***

| **Key Considerations** | **References** |
| --- | --- |
| ISO 3972:2011 - Sensory analysis - Methodology - Method of investigating sensitivity of taste | ISO/TC 34/SC 12 |
| ISO 4120:2021 - Sensory analysis - Methodology - Triangle test | ISO/TC 34/SC 12 |
| ISO 4121:2003 - Sensory analysis - Guidelines for the use of quantitative response scales | ISO/TC 34/SC 12 |
| ISO 5495:2005 - Sensory analysis - Methodology - Paired comparison test | ISO/TC 34/SC 12 |
| ISO 5496:2006 - Sensory analysis - Methodology - Initiation and training of assessors in the detection and recognition of odours | ISO/TC 34/SC 12 |
| ISO 5497:1982 - Sensory analysis - Methodology - Guidelines for the preparation of samples for which direct sensory analysis is not feasible | ISO/TC 34/SC 12 |
| ISO 6658:2017 - Sensory analysis - Methodology - General guidance | ISO/TC 34/SC 12 |
| ISO 8586:2023 - Sensory analysis - Selection and training of sensory assessors | ISO/TC 34/SC 12 |
| ISO 8587:2006 - Sensory analysis - Methodology - Ranking | ISO/TC 34/SC 12 |
| ISO 8588:2017 - Sensory analysis - Methodology - "A" - "not A" test | ISO/TC 34/SC 12 |
| ISO 8589:2007 - Sensory analysis - General guidance for the design of test rooms | ISO/TC 34/SC 12 |
| ISO 10399:2017 - Sensory analysis - Methodology - Duo-trio test | ISO/TC 34/SC 12 |
| ISO 11035:1994 - Sensory analysis - Identification and selection of descriptors for establishing a sensory profile by a multidimensional approach | ISO/TC 34/SC 12 |
| ISO 11036:2020 - Sensory analysis - Methodology - Texture profile | ISO/TC 34/SC 12 |
| ISO 11037:2011 - Sensory analysis - Guidelines for sensory assessment of the colour of products | ISO/TC 34/SC 12 |
| ISO 11056:2021 - Sensory analysis - Methodology - Magnitude estimation method | ISO/TC 34/SC 12 |
| ISO 11132:2021 - Sensory analysis - Methodology - Guidelines for the measurement of the performance of a quantitative descriptive sensory panel | ISO/TC 34/SC 12 |
| ISO 11136:2014 - Sensory analysis - Methodology - General guidance for conducting hedonic tests with consumers in a controlled area | ISO/TC 34/SC 12 |
| ISO 11136:2014/Amd 1:2020 - Sensory analysis - Methodology - General guidance for conducting hedonic tests with consumers in a controlled area - Amendment 1 | ISO/TC 34/SC 12 |
| ISO 13299:2016 - Sensory analysis - Methodology - General guidance for establishing a sensory profile | ISO/TC 34/SC 12 |
| ISO 13300-1:2006 - Sensory analysis- General guidance for the staff of a sensory evaluation laboratory - Part 1: Staff responsibilities | ISO/TC 34/SC 12 |
| ISO 13300-2:2006 - Sensory analysis - General guidance for the staff of a sensory evaluation laboratory - Part 2: Recruitment and training of panel leaders | ISO/TC 34/SC 12 |
| ISO 13301:2018 - Sensory analysis - Methodology - General guidance for measuring odour, flavour and taste detection thresholds by a three-alternative forced-choice (3-AFC) procedure | ISO/TC 34/SC 12 |
| ISO 16779:2015 - Sensory analysis- Assessment (determination and verification) of the shelf life of foodstuffs | ISO/TC 34/SC 12 |
| ISO 16820:2019 - Sensory analysis- Methodology - Sequential analysis | ISO/TC 34/SC 12 |
| ISO 20613:2019 - Sensory analysis - General guidance for the application of sensory analysis in quality control | ISO/TC 87 |
| ISO 22308-1:2021 - Cork bark selected as bottling product - Part 1: Sensory evaluation - Methodology for sensory evaluation by soaking | ISO/TC 34/SC 12 |
| ISO 29842:2024 - Sensory analysis - Methodology - Balanced incomplete block designs | ISO/TC 34/SC 12 |
| Psychophysical methods – Measuring taste and smell thresholds, conducting difference tests and using scales | Kemp et al. (2011c), Kemp et al. (2011d), Lawless and Heymann (2010e), Lawless and Heymann (2010c), Lawless and Heymann (2010d), Lawless and Heymann (2010g) |
| Consumer taste test - assessing food acceptability and preferences through consumers' subjective evaluations | Kemp et al. (2011a), Kemp et al. (2011b), Lawless and Heymann (2010f), Lawless and Heymann (2010a), Lawless and Heymann (2010b) |

***Table 5. Sensory Evaluation Specifics for Space-Related Studies***

| **Key Factor** | **Standardised Methods (Earth Sensory Evaluation)** | **Considerations for Space Environments** |
| --- | --- | --- |
| **Number of Participants** | Minimum 50-100 for consumer tests (affective responses), smaller at 30 for psychophysical evaluation (chemical senses) (Fields, 2013) | Limited crew size (~6 astronauts per mission) – Requires innovative approaches to statistical validity |
| **Type of Stimuli** | Solids, semi-solids, liquids | Solids, semi-solids, liquids |
| **Serving Standards** | Standardised portions, consistent preparation for repeatability | Packaging must account for microgravity (e.g., pouches, tubes); temperature control could be a challenge |
| **Controlled Environments** | Sensory booths, controlled lighting, temperature, and noise levels | Spacecraft constraints on space and conditions; environmental factors (e.g., noise, lighting, pressure) |
| **Bias Prevention** | Blind testing to reduce bias in sensory evaluations | Must consider astronauts’ psychological states (stress, isolation) that may influence sensory perception |
| **Hygiene and Safety Protocols** | Strict protocols for sample handling and serving to avoid contamination | Hygiene is critical in closed-loop life support systems; potential risks in sample storage and preparation |
| **Environmental Conditions Impacting Senses** | Controlled humidity, temperature, airflow for consistency | ISS or spacecraft conditions (air circulation, carbon dioxide levels, odour intensity) could affect perception |
| **Psychophysical Methods Adaptation** | Taste/smell threshold detection, use of scales for subjective ratings | Must adapt to astronaut availability and schedule constraints; fatigue and stress may influence results |

References

Antonutto, G., & Di Prampero, P. (2003). Cardiovascular deconditioning in microgravity: some possible countermeasures. *European journal of applied physiology, 90*(3-4), 283-291.

AWI. (2022, 12.07.2022). *Neumayer Station III*. Retrieved 2.10.2024 from <https://www.awi.de/en/expedition/stations/neumayer-station-iii.html>

Baker, E. S., Barratt, M. R., Sams, C. F., & Wear, M. L. (2019). Human response to space flight. *Principles of clinical medicine for space flight*, 367-411.

Baran, R., Marchal, S., Garcia Campos, S., Rehnberg, E., Tabury, K., Baselet, B., Wehland, M., Grimm, D., & Baatout, S. (2021, Dec 28). The Cardiovascular System in Space: Focus on In Vivo and In Vitro Studies. *Biomedicines, 10*(1). <https://doi.org/10.3390/biomedicines10010059>

Basner, M., Dinges, D. F., Mollicone, D. J., Savelev, I., Ecker, A. J., Di Antonio, A., Jones, C. W., Hyder, E. C., Kan, K., & Morukov, B. V. (2014). Psychological and behavioral changes during confinement in a 520-day simulated interplanetary mission to mars. *PLoS One, 9*(3), e93298.

Beysens, D. A., & Van Loon, J. J. (2015). *Generation and applications of extra-terrestrial environments on earth*. Taylor & Francis.

Bruhaug, G., & Beveridge, L. (2020). Diamagnetic Levitation Using High-Temperature Superconducting Wires for Microgravity Research and Mitigation in Human Spaceflight Applications. *arXiv preprint arXiv:2004.09683*.

Carriot, J., Mackrous, I., & Cullen, K. E. (2021). Challenges to the Vestibular System in Space: How the Brain Responds and Adapts to Microgravity. *Front Neural Circuits, 15*, 760313. <https://doi.org/10.3389/fncir.2021.760313>

Cassady, K., Koppelmans, V., Reuter-Lorenz, P., De Dios, Y., Gadd, N., Wood, S., Castenada, R. R., Kofman, I., Bloomberg, J., Mulavara, A., & Seidler, R. (2016, Nov 1). Effects of a spaceflight analog environment on brain connectivity and behavior. *Neuroimage, 141*, 18-30. <https://doi.org/10.1016/j.neuroimage.2016.07.029>

Childress, S. D., Williams, T. C., & Francisco, D. R. (2023). NASA Space Flight Human-System Standard: enabling human spaceflight missions by supporting astronaut health, safety, and performance. *NPJ Microgravity, 9*(1), 31.

Clement, G. R., Boyle, R. D., George, K. A., Nelson, G. A., Reschke, M. F., Williams, T. J., & Paloski, W. H. (2020, May 1). Challenges to the central nervous system during human spaceflight missions to Mars. *J Neurophysiol, 123*(5), 2037-2063. <https://doi.org/10.1152/jn.00476.2019>

Comfort, P., McMahon, J. J., Jones, P. A., Cuthbert, M., Kendall, K., Lake, J. P., & Haff, G. G. (2021, Oct). Effects of Spaceflight on Musculoskeletal Health: A Systematic Review and Meta-analysis, Considerations for Interplanetary Travel. *Sports Med, 51*(10), 2097-2114. <https://doi.org/10.1007/s40279-021-01496-9>

de Weck, O., & Simchi-Levi, D. (2006). Haughton-Mars project expedition 2005. *Final Report, NASA/TP, 214196*, 2006.

DLR. (n.d). *:envihab - Future Research for Space and Earth*. Retrieved 27.01.2023 from <https://www.dlr.de/envihab/en/>

Douglas, G. L., Cooper, M., Bermudez-Aguirre, D., & Sirmons, T. (2016). *Risk of performance decrement and crew illness due to an inadequate food system*.

Douglas, G. L., Cooper, M. R., Wu, H., Gaza, R., Guida, P., & Young, M. (2021). Impact of galactic cosmic ray simulation on nutritional content of foods. *Life sciences in space research, 28*, 22-25.

Edsall, S. C., & Franz-Odendaal, T. A. (2014). An assessment of the long-term effects of simulated microgravity on cranial neural crest cells in zebrafish embryos with a focus on the adult skeleton. *PLoS One, 9*(2), e89296.

ESA. (n.d-a). *SciSpacE Platforms*. Retrieved 10.10.2023 from <https://scispace.esa.int/scispace-platforms/>

ESA. (n.d-b). *The ZARM drop tower in Bremen*. Retrieved 10.10.2023 from <https://www.esa.int/Education/Drop_Your_Thesis/The_ZARM_drop_tower_in_Bremen>

Ferranti, F., Del Bianco, M., & Pacelli, C. (2020). Advantages and Limitations of Current Microgravity Platforms for Space Biology Research. *Applied Sciences, 11*(1). <https://doi.org/10.3390/app11010068>

Fields, A. (2013). *Discovering statistics using IBM SPSS statistics* (4 ed.). SAGE. <https://books.google.com.au/books?hl=en&lr=&id=c0Wk9IuBmAoC&oi=fnd&pg=PP2&dq=andy+fields+discovering+statistics&ots=LdzmJM_w0A&sig=7vwSqF-xb8AIqw6ep2WgdObg5z8#v=onepage&q=central%20limit%20theorm&f=false>

Franco-Obregón, A., Cambria, E., Greutert, H., Wernas, T., Hitzl, W., Egli, M., Sekiguchi, M., Boos, N., Hausmann, O., & Ferguson, S. J. (2018). TRPC6 in simulated microgravity of intervertebral disc cells. *European Spine Journal, 27*, 2621-2630.

Gemignani, A., Piarulli, A., Menicucci, D., Laurino, M., Rota, G., Mastorci, F., Gushin, V., Shevchenko, O., Garbella, E., & Pingitore, A. (2014). How stressful are 105 days of isolation? Sleep EEG patterns and tonic cortisol in healthy volunteers simulating manned flight to Mars. *International Journal of Psychophysiology, 93*(2), 211-219.

Gonzalez Viejo, C., Harris, N., Tongson, E., & Fuentes, S. (2024b, Oct 9). Exploring consumer acceptability of leafy greens in earth and space immersive environments using biometrics. *NPJ Sci Food, 8*(1), 81. <https://doi.org/10.1038/s41538-024-00314-6>

Goswami, N., White, O., Blaber, A., Evans, J., van Loon, J. J. W. A., & Clement, G. (2021). Human physiology adaptation to altered gravity environments. *Acta Astronautica, 189*, 216-221. <https://doi.org/10.1016/j.actaastro.2021.08.023>

Horstman, A. M., De Ruiter, C., Van Duijnhoven, N., Hopman, M., & De Haan, A. (2012). Changes in muscle contractile characteristics and jump height following 24 days of unilateral lower limb suspension. *European journal of applied physiology, 112*, 135-144.

Hupfeld, K. E., McGregor, H. R., Reuter-Lorenz, P. A., & Seidler, R. D. (2021, Mar). Microgravity effects on the human brain and behavior: Dysfunction and adaptive plasticity. *Neurosci Biobehav Rev, 122*, 176-189. <https://doi.org/10.1016/j.neubiorev.2020.11.017>

Iwase, S., Nishimura, N., Tanaka, K., & Mano, T. (2020). Effects of microgravity on human physiology. *Beyond LEO-Human Health Issues for Deep Space Exploration*.

JAXA. (2008, 23.03.2008). *Astronaut Training Facility (ATF)*. Retrieved 10.05.2024 from <https://iss.jaxa.jp/ssip/ssip_atf_e.html>

Kanas, N. (1990). Psychological, psychiatric, and interpersonal aspects of long-duration space missions. *Journal of spacecraft and rockets, 27*(5), 457-463.

Kanas, N., & Manzey, D. (2008). *Space psychology and psychiatry* (Vol. 16). Springer.

Kemp, S. E., Hollowood, T., & Hort, J. (2011a). Planning your Sensory Project. In *Sensory evaluation: a practical handbook* (pp. 11-29). John Wiley & Sons. <https://doi.org/10.1002/9781118688076>

Kemp, S. E., Hollowood, T., & Hort, J. (2011b). Requirements for Sensory Testing. In *Sensory evaluation: a practical handbook* (pp. 30-65). John Wiley & Sons. <https://doi.org/10.1002/9781118688076>

Kemp, S. E., Hollowood, T., & Hort, J. (2011c). Sensory Perception. In *Sensory evaluation: a practical handbook* (pp. 4-10). John Wiley & Sons. <https://doi.org/10.1002/9781118688076>

Kemp, S. E., Hollowood, T., & Hort, J. (2011d). Sensory Test Methods. In *Sensory evaluation: a practical handbook* (pp. 66-137). John Wiley & Sons. <https://doi.org/10.1002/9781118688076>

Lackner, J. R., & Graybiel, A. (1979). Parabolic flight: loss of sense of orientation. *Science, 206*(4422), 1105-1108.

Lawless, H. T., & Heymann, H. (2010a). Acceptance Testing. In *Sensory evaluation of food: principles and practices* (pp. 325-344). Springer Science & Business Media.

Lawless, H. T., & Heymann, H. (2010b). Consumer Field Tests and Questionnaire Design. In *Sensory evaluation of food: principles and practices* (pp. 349-378). Springer Science & Business Media.

Lawless, H. T., & Heymann, H. (2010c). Discrimination Testing. In *Sensory evaluation of food: principles and practices* (pp. 79-99). Springer Science & Business Media.

Lawless, H. T., & Heymann, H. (2010d). Measurement of Sensory Thresholds. In *Sensory evaluation of food: principles and practices* (pp. 125-145). Springer Science & Business Media.

Lawless, H. T., & Heymann, H. (2010e). Physiological and Psychological Foundations of Sensory Function. In *Sensory evaluation of food: principles and practices* (pp. 19-50). Springer Science & Business Media.

Lawless, H. T., & Heymann, H. (2010f). Preference Testing. In *Sensory evaluation of food: principles and practices* (pp. 303-323). Springer Science & Business Media.

Lawless, H. T., & Heymann, H. (2010g). Scaling. In *Sensory evaluation of food: principles and practices* (pp. 149-174). Springer Science & Business Media.

Leach, C., Johnson, P., & Cintron, N. (1988). The endocrine system in space flight. *Acta Astronautica, 17*(2), 161-166.

Lee, A. G., Mader, T. H., Gibson, C. R., Tarver, W., Rabiei, P., Riascos, R. F., Galdamez, L. A., & Brunstetter, T. (2020). Spaceflight associated neuro-ocular syndrome (SANS) and the neuro-ophthalmologic effects of microgravity: a review and an update. *NPJ Microgravity, 6*(1), 7.

Lee, P. (2002). Mars on Earth: The NASA Haughton-Mars Project. *Ad Astra: The Magazine of the National Space Society, 14*(3).

Loke, G., Chandrapala, J., Besnard, A., Kantono, K., Brennan, C., Newman, L., & Low, J. (2024b). Food odour perception and affective response in Virtual spacecraft and microgravity body posture (1-G) – Potential ground-based simulations. *Food Research International*. <https://doi.org/10.1016/j.foodres.2024.115260>

Marmolejo, J., & Ewert, M. (2016). Human Exploration System Test-Bed for Integration and Advancement (HESTIA) Support of Future NASA Deep-Space Missions. Annual AIAA Technical Symposium of the Houston Section,

MEDES. (2023). *Space Clinic*. <https://www.medes.fr/en/space-clinic/equipment/>

Milon, H., Decarli, B., Adine, A.-M., & Kihm, E. (1996). Food intake and nutritional status during EXEMSI. In *Advances in space biology and medicine* (Vol. 5, pp. 79-91). Elsevier.

NASA. (2023a). *About Sounding Rockets*. Retrieved 10.10.2023 from <https://www.nasa.gov/soundingrockets/overview/>

NASA. (2023b). *Analog Missions*. Retrieved 17.11.2023 from <https://www.nasa.gov/analog-missions/>

NASA. (2024a, Jul 02, 2024). *Mission Success: HERA Crew Successfully Completes 45-Day Simulated Journey to Mars*. Retrieved 2.10.2024 from <https://www.nasa.gov/centers-and-facilities/johnson/mission-success-hera-crew-successfully-completes-45-day-simulated-journey-to-mars/>

NASA. (2024b, 14.06.2024). Houston We Have a Podcast In *NBL Environments*. <https://www.nasa.gov/podcasts/houston-we-have-a-podcast/nbl-environments/>

NASA. (2024c). *NEK and SIRIUS*. <https://www.nasa.gov/mission/nek-and-sirius/>

Norsk, P. (2020). Adaptation of the cardiovascular system to weightlessness: Surprises, paradoxes and implications for deep space missions. *Acta Physiologica, 228*(3), e13434. <https://doi.org/https://doi.org/10.1111/apha.13434>

Noskov, V. B. (2013). Redistribution of bodily fluids under conditions of microgravity and in microgravity models. *Human Physiology, 39*(7), 698-706. <https://doi.org/10.1134/s0362119713070128>

NSF. (2024). *United States Antarctic Program*. <https://www.usap.gov/aboutthecontinent/>

Olabi, A. A., Lawless, H. T., Hunter, J. B., Levitsky, D. A., & Halpern, B. P. (2002, Mar). The effect of microgravity and space flight on the chemical senses. *J Food Sci, 67*(2), 468-478. <https://doi.org/10.1111/j.1365-2621.2002.tb10622.x>

Oluwafemi, F. A., & Neduncheran, A. (2022). Analog and simulated microgravity platforms for life sciences research: Their individual capacities, benefits and limitations. *Advances in Space Research, 69*(7), 2921-2929. <https://doi.org/10.1016/j.asr.2022.01.007>

Ong, J., Mader, T. H., Gibson, C. R., Mason, S. S., & Lee, A. G. (2023). Spaceflight associated neuro-ocular syndrome (SANS): an update on potential microgravity-based pathophysiology and mitigation development. *Eye*, 1-7.

Pagel, J. I., & Choukèr, A. (2016). Effects of isolation and confinement on humans-implications for manned space explorations. *Journal of Applied Physiology*.

Palinkas, L. A. (1992). Going to extremes: the cultural context of stress, illness and coping in Antarctica. *Social Science & Medicine, 35*(5), 651-664.

Palinkas, L. A., Johnson, J. C., & Boster, J. S. (2004). Social support and depressed mood in isolated and confined environments. *Acta Astronautica, 54*(9), 639-647.

Palma Jiménez, M., Corrales Ureña, Y. R., Villalobos Bermúdez, C., & Vega Baudrit, J. (2017). Microgravity and Nanomaterials.

Schmitt, D. A., & Schaffar, L. (1993). Isolation and confinement as a model for spaceflight immune changes. *Journal of Leucocyte Biology, 54*(3), 209-213.

Schneider, S., Brümmer, V., Carnahan, H., Kleinert, J., Piacentini, M. F., Meeusen, R., & Strüder, H. K. (2010). Exercise as a countermeasure to psycho-physiological deconditioning during long-term confinement. *Behavioural brain research, 211*(2), 208-214.

Schneider, S., Cheung, J. J., Frick, H., Krehan, S., Micke, F., Sauer, M., Dalecki, M., & Dern, S. (2014). When neuroscience gets wet and hardcore: neurocognitive markers obtained during whole body water immersion. *Experimental brain research, 232*, 3325-3331.

Seitz, S. J., & Glass, B. J. (2017). Haughton-Mars Project at 20: Challenges and Designs for Future Exploration. Congress on Technical Advancement 2017,

Šolcová, I. P., Šolcová, I., Stuchlíková, I., & Mazehóová, Y. (2016). The story of 520 days on a simulated flight to Mars. *Acta Astronautica, 126*, 178-189. <https://www.sciencedirect.com/science/article/pii/S0094576515301983>

Strollo, F., Macchi, C., Eberini, I., Masini, M. A., Botta, M., Vassilieva, G., Nichiporuk, I., Monici, M., Santucci, D., & Celotti, F. (2018). Body composition and metabolic changes during a 520-day mission simulation to Mars. *Journal of Endocrinological Investigation, 41*, 1267-1273.

Stuster, J. (2016). *Behavioral issues associated with long-duration space expeditions: review and analysis of astronaut journals: experiment 01-E104 (Journals)*. National Aeronautics and Space Administration, Johnson Space Center Houston, TX.

Stuster, J. W. (2007). Bold endeavors: behavioral lessons from polar and space exploration. *Gravitational and Space Research, 13*(2).

Veronika, C., Tatiana, B., Galina, S., Andrej, F., & Sergei, M. (2019). 3D-clinorotation induces specific alterations in metabolite profiles of germinating Brassica napus L. seeds. *Biological Communications, 64*(1), 55-74.

Vessel, E. A., & Russo, S. (2015). Effects of reduced sensory stimulation and assessment of countermeasures for sensory stimulation augmentation. *NASA Rep*.

Watkins, P., Hughes, J., Gamage, T. V., Knoerzer, K., Ferlazzo, M. L., & Banati, R. B. (2022). Long term food stability for extended space missions: a review. *Life sciences in space research, 32*, 79-95.

Wilson, J. M., Krigsfeld, G. S., Sanzari, J. K., Wagner, E. B., Mick, R., & Kennedy, A. R. (2012). Comparison of hindlimb unloading and partial weight suspension models for spaceflight-type condition induced effects on white blood cells. *Advances in Space Research, 49*(2), 237-248.

Yang, J. Q., Jiang, N., Li, Z. P., Guo, S., Chen, Z. Y., Li, B. B., Chai, S. B., Lu, S. Y., Yan, H. F., Sun, P. M., Zhang, T., Sun, H. W., Yang, J. W., Zhou, J. L., Yang, H. M., & Cui, Y. (2020, Nov). The effects of microgravity on the digestive system and the new insights it brings to the life sciences. *Life Sci Space Res (Amst), 27*, 74-82. <https://doi.org/10.1016/j.lssr.2020.07.009>

Zwart, S., Kloeris, V., Perchonok, M., Braby, L., & Smith, S. (2009). Assessment of nutrient stability in foods from the space food system after long‐duration spaceflight on the ISS. *Journal of food science, 74*(7), H209-H217.

Zwart, S. R., Mulavara, A. P., Williams, T. J., George, K., & Smith, S. M. (2021). The role of nutrition in space exploration: Implications for sensorimotor, cognition, behavior and the cerebral changes due to the exposure to radiation, altered gravity, and isolation/confinement hazards of spaceflight. *Neuroscience & Biobehavioral Reviews, 127*, 307-331.
